# TRAF3 loss protects glioblastoma cells from lipid peroxidation and immune elimination via dysregulated lipid metabolism

**DOI:** 10.1172/JCI178550

**Published:** 2025-02-11

**Authors:** Yu Zeng, Liqian Zhao, Kunlin Zeng, Ziling Zhan, Zhengming Zhan, Shangbiao Li, Hongchao Zhan, Peng Chai, Cheng Xie, Shengfeng Ding, Yuxin Xie, Li Wang, Cuiying Li, Xiaoxia Chen, Daogang Guan, Enguang Bi, Jianyou Liao, Fan Deng, Xiaochun Bai, Ye Song, Aidong Zhou

**Affiliations:** 1 Department of Cell Biology, School of Basic Medical Science, Southern Medical University, Guangzhou, China.; 2 Department of Neurosurgery, Shanghai Ninth People’s Hospital, Shanghai Jiao Tong University School of Medicine, Shanghai, China.; 3 Department of Neurosurgery, Nanfang Hospital, Southern Medical University, Guangzhou, China.; 4 Guangdong Provincial Key Laboratory of Malignant Tumor Epigenetics and Gene Regulation, Research Center of Medicine, Sun Yat-sen Memorial Hospital, Sun Yat-sen University, Guangzhou, China.; 5 Department of Radiation Oncology, Zhujiang Hospital, Southern Medical University, Guangzhou, China.; 6 Department of Biochemistry and Molecular Biology, School of Basic Medical Sciences, Southern Medical University, Guangzhou, China.; 7 Department of Neurosurgery, Guangzhou Women and Children’s Medical Center, Guangzhou Medical University, Guangdong Provincial Clinical Research Center for Child Health, Guangzhou, China.; 8 Guangdong Province Key Laboratory of Molecular Tumor Pathology, School of Basic Medical Sciences, Southern Medical University, Guangzhou, China.

**Keywords:** Cell biology, Metabolism, Brain cancer, Cancer immunotherapy, Fatty acid oxidation

## Abstract

Glioblastoma (GBM) is a highly aggressive form of brain tumor characterized by dysregulated metabolism. Increased fatty acid oxidation (FAO) protects tumor cells from lipid peroxidation–induced cell death, although the precise mechanisms involved remain unclear. Here, we report that loss of TNF receptor–associated factor 3 (TRAF3) in GBM critically regulated lipid peroxidation and tumorigenesis by controlling the oxidation of polyunsaturated fatty acids (PUFAs). TRAF3 was frequently repressed in GBM due to promoter hypermethylation. TRAF3 interacted with enoyl-CoA hydratase 1 (ECH1), an enzyme that catalyzes the isomerization of unsaturated FAs (UFAs) and mediates K63-linked ubiquitination of ECH1 at Lys214. ECH1 ubiquitination impeded TOMM20-dependent mitochondrial translocation of ECH1, which otherwise promoted the oxidation of UFAs, preferentially the PUFAs, and limited lipid peroxidation. Overexpression of TRAF3 enhanced the sensitivity of GBM to ferroptosis and anti–programmed death–ligand 1 (anti–PD-L1) immunotherapy in mice. Thus, the TRAF3/ECH1 axis played a key role in the metabolism of PUFAs and was crucial for lipid peroxidation damage and immune elimination in GBM.

## Abstract 2

Zeng* et. al.* demonstrate that TRAF3 loss promotes ECH1-mediated oxidation of polyunsaturated fatty acid (PUFA) and protects glioblastoma cells from lipid peroxidation damage. Overexpression of TRAF3 enhances the responsiveness of glioblastoma to ferropotosis and anti-PD-L1 immunotherapy.

## Graphical abstract

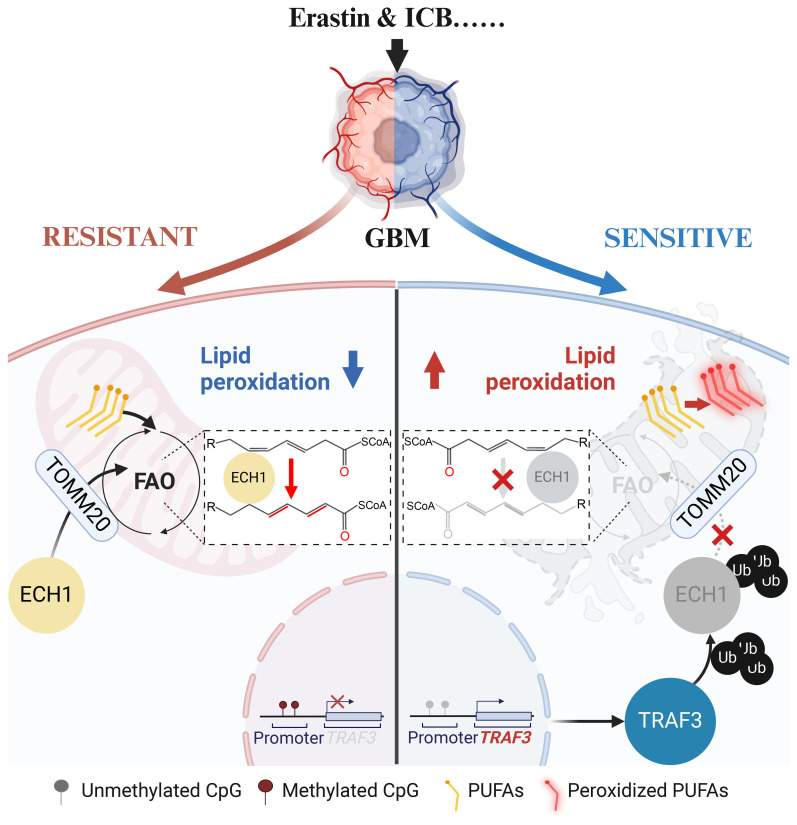

## Introduction

Glioblastoma (GBM), a highly aggressive type of brain cancer, is notorious for its resistance to treatments and poor prognosis, with a median overall survival of only 15–18 months (1, 2). One hallmark of GBM is the reprogramming of cellular metabolism, allowing cancer cells to acquire increased energy and materials for their survival and growth (3, 4). Consequently, while GBM relies on glycolysis for energy, it exhibits a high degree of metabolic flexibility, enabling it to modulate its metabolic programs in response to the heterogeneity of the tumor microenvironment, including hypoxia, nutrient deprivation, and oxidative stress (5). In recent years, fatty acid oxidation (FAO) has gained significant attention, given its critical contribution to the metabolic plasticity of GBM cells. FAO has been identified as an important source of ATP for highly glycolytic mesenchymal GBM cells to survive in nutrient-restricted conditions, and targeting FAO has emerged as a promising therapeutic approach for the treatment of GBM (6, 7).

Unsaturated FAs (UFAs) constitute an important source of lipids, which are required for the maintenance of membrane fluidity, signal transduction, and the lipid pool for β-oxidation (8). FA desaturases are enzymes that catalyze the addition of double carbon bonds to acyl chains, a vital process in the formation of mono- and polyunsaturated FAs (MUFAs and PUFAs, respectively) (9). In recent years, the composition of membrane FAs, particularly the ratios of saturated FAs (SFAs), MUFAs, and PUFAs in regulating cell survival and lipotoxicity-related ferroptosis has been increasingly appreciated (10–12). However, although most studies focused on the biosynthesis of UFAs, less attention has been paid to the regulation of UFA oxidation.

TNF receptor–associated factors (TRAFs) (TRAF1–7) constitute a family of intracellular signaling adaptors that interact with a range of receptors, including TNFRs, TLRs, and IL receptors and play crucial roles in innate immune signaling (13, 14). As a member of the TRAF family, TRAF3 is expressed in a variety of immune and nonimmune cell types in mammals (15). TRAF3-KO mice die soon after birth, indicating that TRAF3 possesses an essential and unique function that cannot be substituted by other TRAF members (16). Through its scaffolding function and E3 ubiquitin ligase activity, TRAF3 differentially modulates downstream signaling cascades, such as activation of NF-κB, MAPKs, and IFN-regulatory factors (IRFs) (15, 17). Although the functions of TRAF3 in innate and adaptive immunity are well understood, its roles in tumor malignancy are less explored.

In this study, we investigated the prognostic value of the TRAF family members in cancer and identified TRAF3 as a member frequently repressed in GBM due to promoter hypermethylation. We found that TRAF3-mediated K63 ubiquitination of enoyl-CoA hydratase 1 (ECH1), an enzyme catalyzing the isomerization and metabolism of UFAs (18), impeded ECH1 mitochondrial translocation and critically regulated the oxidation of PUFAs and lipid peroxidation. Exogenous expression of TRAF3 enhanced the sensitivity of GBM to ferroptosis and effectively synergized with anti–programmed death–ligand 1 (anti–PD-L1) therapy in an orthotopic GBM model, indicating a promising therapeutic strategy for glioblastoma.

## Results

### Loss of TRAF3 by promoter hypermethylation correlates with a poor prognosis in glioma.

**Figure 1:**
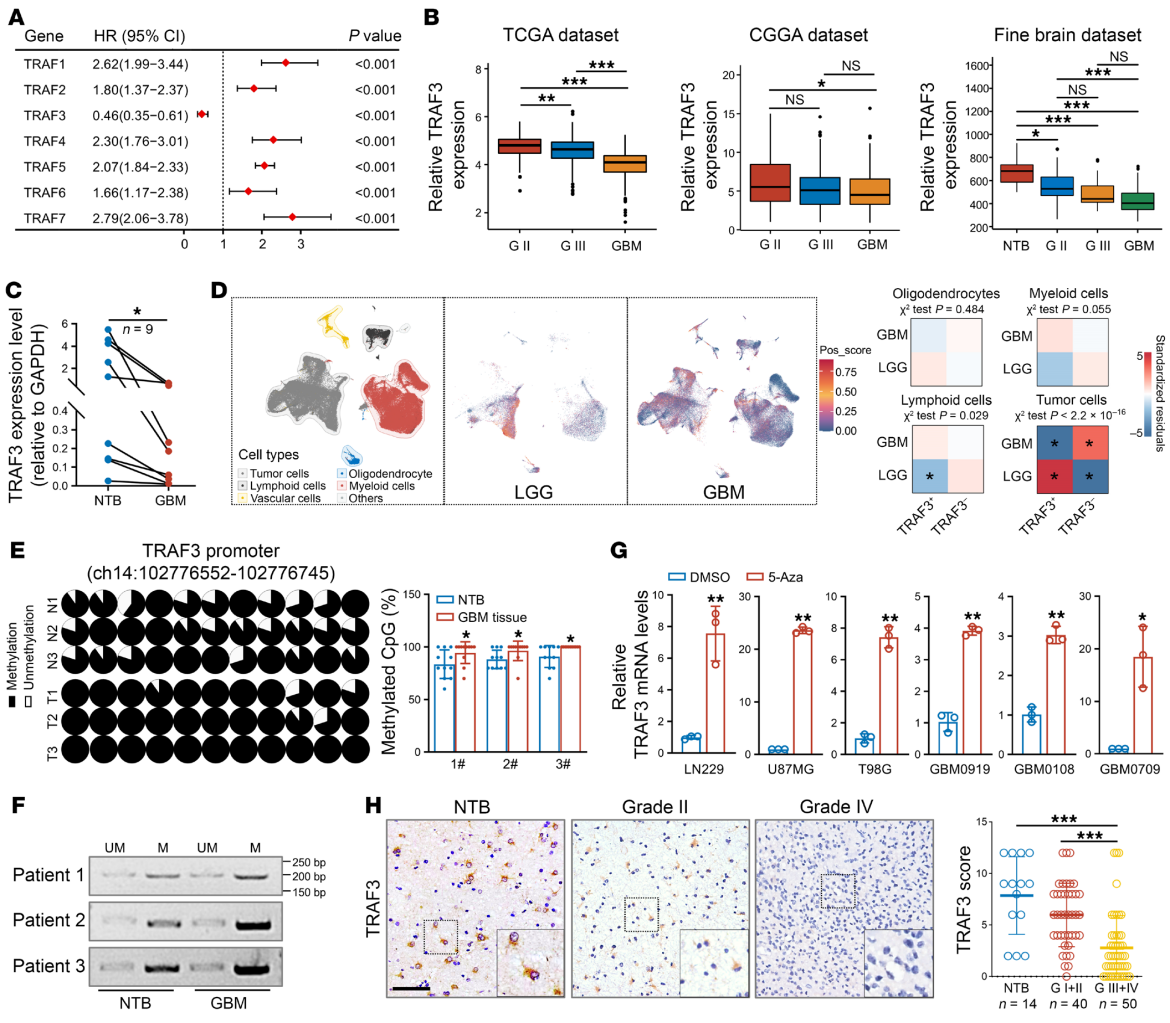
Loss of *TRAF3* by hypermethylation correlates with poor prognosis in glioma. (**A**) Multivariate cox regression analysis of the TRAF family members in TCGA-glioma cohort. (**B**) Analysis of *TRAF3* expression in glioma using TCGA, CGGA, and the Fine brain datasets, respectively. *P < 0.05, **P < 0.01, and ***P < 0.001. (**C**) Analysis of *TRAF3* expression by qRT-PCR in GBM and paired nontumor brain (NTB) tissues (*n* = 9). *P < 0.05. (**D**) Analysis of single-cell RNA-Seq data showing the expression of *TRAF3* across different cell types in low-grade glioma (LGG) and GBM (SCP1985), respectively. The *P* values are indicated. Pos, positive. (**E**) BSP-PCR was used to analyze methylation of the *TRAF3* promoter in GBM tissues and paired NTB tissues. The percentage of methylated CpG was statistically analyzed (mean ± SD, *n* = 11 CpG sites for each tissue). *P < 0.05. (**F**) MSP analysis of 3 pairs of NTB and GBM tissues. UM, unmethylated; M, methylated. (**G**) GBM cells were treated with DMSO or 5-azacytidine (5-Aza), and *TRAF3* mRNA levels were analyzed by qRT-PCR. *GAPDH* was used as an internal control. Values were normalized to DMSO (mean ±SD, *n* = 3 independent experiments). *P < 0.05 and **P < 0.01. (**H**) TRAF3 protein expression in different grades of gliomas and NTB tissues was analyzed by immunostaining. Representative images are shown. Scale bar: 200 μm. Original magnification, ×200 (insets). Staining for TRAF3 was scored on a scale of 0–12, and the expression scores for TRAF3 in grades III+IV were compared with those for NTB and grades I+II (*n* = 14 NTB, *n* = 40 grades I+II, and *n* = 50 grades III+IV). ***P < 0.001. Statistical analysis was performed using 1-way ANOVA with Tukey’s post hoc test (**B** and **H**), paired, 2-tailed Student’s *t* test (**C**), χ^2^ test (**D**), or unpaired, 2-tailed Student’s *t* test (**E** and **G**). G II, grade II; G III, grade III.

Although the TRAF family members as mediators of innate immune receptor signaling have been extensively investigated in myeloid cells (15), their roles in tumor malignancy have been less extensively explored. To comprehensively evaluate the prognostic significance of TRAF family members (TRAF1–7) in glioma, we first analyzed their expression patterns in The Cancer Genome Atlas (TCGA) glioma cohort. While high expression of other members was associated with a poor prognosis, high-level *TRAF3* correlated with significantly longer overall survival of patients with glioma (Figure 1A). Interestingly, pan-cancer survival analysis demonstrated that glioma was the only cancer type in which *TRAF3* levels negatively correlated with patient survival (Supplemental Figure 1A; supplemental material available online with this article; https://doi.org/10.1172/JCI178550DS1), highlighting the distinctive role of TRAF3 in glioma compared with other tumor types. In multiple glioma datasets including TCGA-glioma, the Chinese Glioma Genome Atlas (CGGA-glioma), and the Fine brain dataset, a decrease in *TRAF3* levels was observed as the tumor grade advanced (Figure 1B). The reduction in *TRAF3* mRNA levels observed in GBM was further validated using paired tumor and nontumor brain tissues through quantitative reverse transcription PCR (qRT-PCR) (Figure 1C).

It has been previously documented that TRAF3 is predominantly expressed in glial cells, neuronal cells, and immune cells, including T and B cells (19). To examine the cell-type–specific expression pattern of *TRAF3* in glioma, we conducted single-cell RNA-Seq analysis based on publicly available datasets. In low-grade glioma (LGG), high *TRAF3* expression was observed in tumor cells, while moderate expression was noted in myeloid cells (Figure 1D). In contrast, there was a notable reduction in *TRAF3* expression in GBM tumor cells relative to those in LGG tumor cells. Notably, although *TRAF3* expression was higher in myeloid and lymphoid cells in GBM compared with LGG (Figure 1D), the bulk RNA-Seq results indicated lower overall *TRAF3* expression in the GBM cells (Supplemental Figure 1B). This discrepancy suggests that *TRAF3* expression in tumor cells, as opposed to nontumor cells, is the primary determinant of overall TRAF3 levels in glioma. To elucidate the potential value of *TRAF3* in prognosis, we performed a multivariate Cox regression analysis involving *TRAF3* expression, O6-methylguanine-DNA methyltransferase (MGMT) methylation status, isocitrate dehydrogenase (IDH) mutation status, sex, grade, and age. The results demonstrated that among these factors, TRAF3 remained an independent predictor of a favorable prognosis in glioma, with elevated TRAF3 expression consistently correlating with improved survival outcomes, regardless of other clinical and genetic variables (Supplemental Figure 1C). These findings underscore the potential of TRAF3 as a valuable biomarker for glioma grading and indicate its specific involvement in glioma development.

Hypermethylation of the *TRAF3* promoter has been observed in cervical precancerous lesions compared with healthy controls (20). Moreover, a recent study indicates that methylation sites frequently occur in CpG shores, regions approximately 0–2 kb away from the CpG islands in GBM (21). Consistent with this, our analysis revealed a higher probability of DNA methylation in the CpG shore within the *TRAF3* promoter region (Supplemental Figure 1D), implying that promoter methylation may serve as a critical mechanism for *TRAF3* expression. To validate our findings, we performed bisulfite sequencing PCR (BSP-PCR) and methylation-specific PCR (MSP) assays to analyze the promoter methylation status of *TRAF3*. Our results demonstrated that the proportion of *TRAF3* promoter methylation was significantly higher in GBM tissues than in the paired nontumor brain tissues (Figure 1, E and F). Furthermore, treatment with 5-azacytidine (5-Aza), a DNA methyltransferase inhibitor, resulted in a significant upregulation of TRAF3 mRNA and protein levels in both patient-derived and commercially available GBM cell lines (Figure 1G and Supplemental Figure 1E). Accordingly, we detected high *TRAF3* promoter methylation levels in GBM cells (Supplemental Figure 1F). Consistent with the mRNA data, TRAF3 protein levels were markedly diminished in glioma specimens relative to levels in nontumor tissues and negatively correlated with the glioma grade (Figure 1H). Taken together, these results demonstrate that TRAF3 was repressed in high-grade glioma by promoter hypermethylation and that decreased TRAF3 level correlated with poor survival of patients with glioma.

### TRAF3 promotes ROS-related mitochondrial damage and inhibits GBM tumorigenesis.

**Figure 2:**
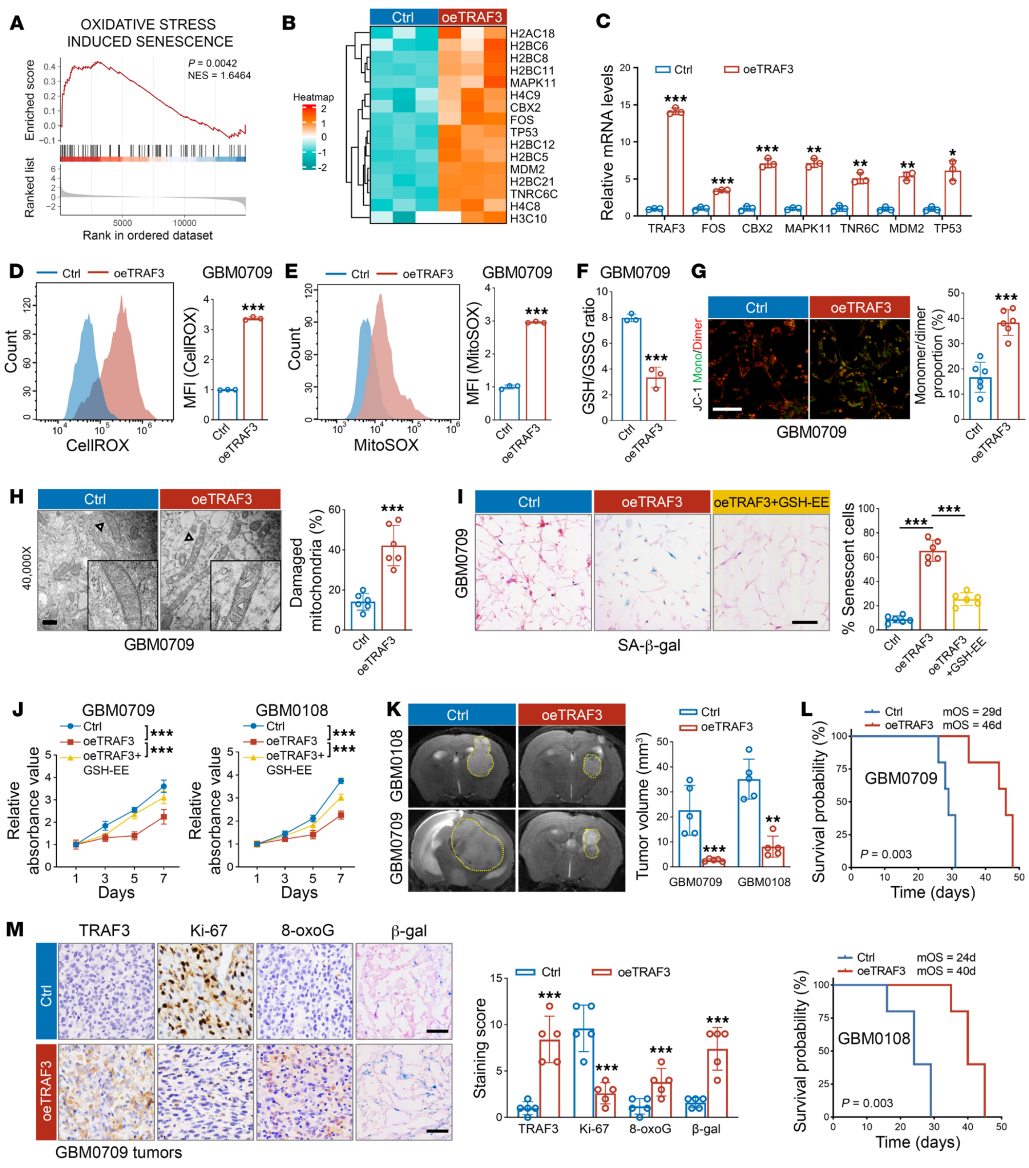
TRAF3 promotes ROS-induced mitochondrial damage and inhibits GBM tumorigenesis. (**A**) GSEA was performed to discern gene expression changes following TRAF3 overexpression (oeTRAF3) in GBM0709 cells. (**B**) Oxidative stress–related gene heatmap in oeTRAF3 GBM0709 cells. (**C**) qRT-qPCR of selected genes in oeTRAF3 cells, normalized to GAPDH (*n* = 3). (**D** and **E**) Flow cytometry of cellular (CellROX) and mitochondrial ROS (MitoSOX) median fluorescence intensity (MFI) in oeTRAF3 cells (*n* = 3). (**F**) GSH/GSSG ratio in oeTRAF3 cells (*n* = 3). (**G**) JC-1 staining of oeTRAF3 cells. Scale bar: 100 μm. The monomer/dimer ratios of JC-1 were statistically analyzed (*n* = 6). (**H**) TEM images of mitochondria in oeTRAF3 cells. Scale bar: 500 nm (original magnification, ×40,000; enlarged magnification, ×80,000). The proportion of damaged mitochondria was statistically analyzed (*n* = 6). (**I**) SA-β-gal^+^ cell percentages in oeTRAF3 cells treated or not with GSH-EE (*n* = 6). (**J**) Cell viability of TRAF3-OE GBM0709 or GBM0108 cells treated or not with GSH-EE by cell counting kit-8 (CCK8). Absorbance values were normalized to the control (*n* = 4). (**K**) GBM0709 or GBM0108 cells (5 × 10^5^ cells/mouse) stably expressing TRAF3 were intracranially (i.c.) injected into nude mice, and tumor growth was monitored by MRI. Tumor volumes in each group were statistically analyzed (*n* = 5). (**L**) The survival of mice bearing GBM0709 and GBM0108 GBM tumors was evaluated (*n* = 5). (**M**) Consecutive mouse GBM tissues derived from GBM0709 cells were stained for TRAF3, Ki-67, 8-oxoG, and β-gal, respectively. Scale bars: 100 μm. Staining scores were compared (*n* = 5). Statistical significance was determined using an unpaired, 2-tailed Student’s *t* test (**C**–**H**, **K**, and **M**), 1-way ANOVA with Tukey’s post hoc test (**I** and **J**), or a log-rank test (**L**). Ctrl, control. Repeated data are presented as the mean ± SD. *P < 0.05, **P < 0.01, and ***P < 0.001.

To explore the functional implications of TRAF3 loss in glioma pathogenesis, we induced overexpression of TRAF3 in patient-derived GBM0709 cells and then profiled gene expression by transcriptome sequencing (Supplemental Table 1). Gene set enrichment analysis (GSEA) revealed a notable enrichment in the gene set associated with oxidative stress–induced senescence (Figure 2A and Supplemental Table 2). Following overexpression of TRAF3, a panel of oxidative stress–related genes, including *TP53*, *MDM2*, and *FOS*, showed a marked increase in expression (Figure 2B). Furthermore, we observed a global alteration in the expression of histone genes, which have been demonstrated to be involved in oxidative stress–induced senescence (22). Some of those genes were further validated by qRT-PCR (Figure 2C).

As previous studies have indicated that TRAF3 plays a role in regulating the oxidative status in cardiomyocytes (23), we proceeded to investigate the effect of TRAF3 on the production of ROS and cellular senescence in GBM. The results demonstrated that both cellular and mitochondrial ROS were markedly elevated in GBM0709 and GBM0108 cells following TRAF3 overexpression (Figure 2, D and E, and Supplemental Figure 2, A and B). Concurrently, the ratio of reduced glutathione (GSH) to oxidized glutathione (GSSG) (GSH/GSSG) was decreased (Figure 2F and Supplemental Figure 2C). Given the close association between mitochondrial ROS levels and mitochondrial stability and function, we further examined the effect of TRAF3 on mitochondrial function. We observed a substantial decrease in the mitochondrial membrane potential of GBM cells following TRAF3 overexpression, as evidenced by JC-1 staining (Figure 2G and Supplemental Figure 2D). Transmission electron microscopy (TEM) images further demonstrated structural damage to the mitochondria, characterized by swollen cristae, upon TRAF3 overexpression (Figure 2H).

Senescence is a typical cellular phenomenon that is induced by the overproduction of cellular ROS and is characterized by irreversible cell-cycle arrest (24). Consistent with the results of transcriptome sequencing, we found that TRAF3 overexpression led to a significant increase in senescent cells and in the growth arrest of GBM cells, both of which were reversed by the addition of exogenous glutathione ethyl ester (GSH-EE) (Figure 2, I and J, and Supplemental Figure 2, E and F). Moreover, the expression of senescence markers, including clusterin, p53, and p21, was markedly elevated following TRAF3 overexpression (Supplemental Figure 2G). Using an in vivo intracranial mouse model, we further explored the role of TRAF3 in GBM tumorigenesis. We found that TRAF3 overexpression substantially suppressed GBM tumorigenicity in vivo and prolonged the overall survival of mice bearing GBM0709 tumors (median survival duration of 29 days for control vs. 46 days for TRAF3-overexpressing [oeTRAF3] mice) and GBM0108 tumors (24 days vs. 40 days) (Figure 2, K and L). Immunostaining of mouse GBM tissues demonstrated that TRAF3 overexpression significantly decreased the levels of Ki-67, whereas the levels of 8-oxoguanine (8-oxoG), indicative of oxidative DNA damage, and β-gal were increased (Figure 2M and Supplemental Figure 2H). Together, these findings suggest that TRAF3-induced ROS production and mitochondrial damage inhibited GBM tumorigenesis.

### TRAF3 interacts with and ubiquitinates ECH1 at Lys214 through K63-linked ubiquitin chains.

**Figure 3:**
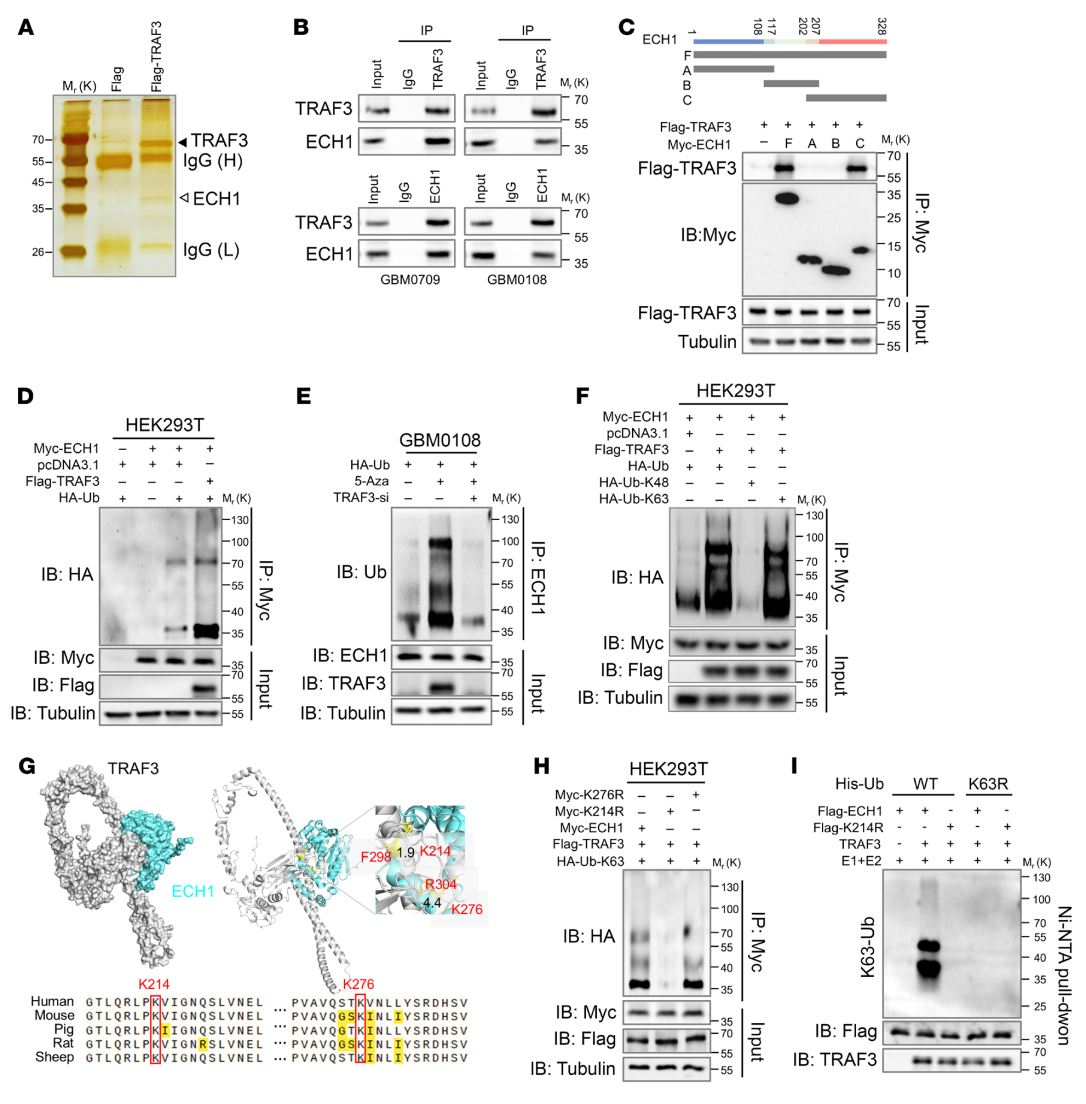
TRAF3 interacts with ECH1 and promotes K63-linked ubiquitination of ECH1 at Lys214. (**A**) U87MG cells were transfected with Flag-TRAF3, and cell lysates were incubated with an anti–Flag-Tag antibody. The immunoprecipitated protein complexes were resolved by SDS-PAGE, and specific protein bands, identified by silver staining, were subjected to MS analysis. The black arrowhead indicates the TRAF3 band, and the white arrowhead indicates the ECH1 band identified by tandem MS/MS. IgH and IgL indicate the IgG heavy chain and light chain, respectively. H, heavy chain; L, light chain. (**B**) Coimmunoprecipitation assays demonstrate the reciprocal interaction between TRAF3 and ECH1 in GBM0709 and GBM0108 cells after treatment with 5-Aza. (**C**) Myc-tagged full-length ECH1 or deletion mutants were cotransfected with Flag-TRAF3 into HEK293T cells. Cell lysates were immunoprecipitated using an anti-Myc antibody, and the immunoprecipitates were analyzed by immunoblotting (IB) using the indicated antibodies. (**D**) HEK293T cells were transfected with the Myc-ECH1, Flag-TRAF3, and HA-Ub plasmids. The cell lysates were incubated with an anti–Myc-tag antibody and then subjected to immunoblotting. (**E**) GBM0108 cells were transfected with TRAF3 siRNA and HA-ubiquitin (HA-Ub), and then were treated with 5-Aza. The cell lysates were immunoprecipitated with an anti-Myc antibody and then analyzed by immunoblotting. (**F**) HEK293T cells were transfected with the indicated plasmids and then incubated with an anti–Myc-tag antibody. The resultant immunoprecipitates were analyzed by immunoblotting. (**G**) The binding mode between TRAF3 and ECH1 was predicted by the HDOCK server. The protein tertiary structures for TRAF3 and ECH1 were retrieved from the Alphafold database. Yellow dashed lines indicate possible interactions, and residues highlighted in red indicate potential residues mediating protein-protein interactions. The interatomic distances between TRAF3 and ECH1 are indicated. (**H**) HEK293T cells were transfected with Flag-TRAF3, HA-Ub-K63, and Myc-ECH1 or each of the ECH1 mutants (K214R or K276R). The cell lysates were immunoprecipitated with an anti-Myc antibody and then analyzed by immunoblotting. (**I**) In vitro ubiquitination assay using the indicated purified proteins in the presence of E1 and E2 enzymes and Mg^2+^-ATP (10 mM) in the ubiquitination reaction buffer. The reaction mixtures were purified by Ni-NTA beads, and the eluted proteins were analyzed by immunoblotting. M, molecular weight; K, kDa.

To explore the underlying mechanism by which TRAF3 regulates oxidative mitochondrial damage, we sought to identify the interacting proteins of TRAF3. To this end, we immunoprecipitated Flag-tagged TRAF3 expressed in U87MG cells and identified the interacting proteins through silver staining and mass spectrometry (MS) analysis (Supplemental Table 3). Among the identified proteins with high abundance, ECH1, an enzyme that catalyzes the isomerization of unsaturated FAs for subsequent oxidation (18, 25), was selected for further investigation (Figure 3A). We confirmed the reciprocal cellular interaction between TRAF3 and ECH1 in GBM cells following treatment with 5-Aza and in HEK293T cells after expression of the exogenously tagged proteins (Figure 3B and Supplemental Figure 3A). To determine the specific protein region of ECH1 that mediates its interaction with TRAF3, we constructed plasmids expressing different truncation mutants of ECH1 (Figure 3C). Coimmunoprecipitation results revealed that the C-terminal region of ECH1 (amino acids 202–328) was indispensable for its interaction with TRAF3 (Figure 3C).

The TRAF family members have been extensively studied as E3 ligases for substrate recognition and ubiquitination, especially in antiviral innate immunity (26), although only a few TRAF3 ubiquitination targets have been identified. We next determined the effect of TRAF3 on ECH1 ubiquitination. We found that TRAF3 overexpression increased the ubiquitination level of ECH1 in HEK293T cells, and we confirmed the result in GBM cells (Figure 3D and Supplemental Figure 3B). Accordingly, depletion of TRAF3 in SW1783 cells, a LGG cell line with relatively high TRAF3 expression, resulted in a marked reduction in ECH1 ubiquitination (Supplemental Figure 3C). Notably, treatment with 5-Aza, which upregulated TRAF3 expression, increased ECH1 ubiquitination in GBM0108 cells, and this effect was reversed by further silencing of TRAF3 (Figure 3E). Because TRAF3 mediates both K48- and K63-linked ubiquitination (27, 28), we next determined the chain preference of ECH1 ubiquitination by TRAF3. The results demonstrated that, while the WT and the K63-mutant ubiquitin (Ubi-K63, K63 WT only) strongly induced ECH1 ubiquitination, the K48-mutant ubiquitin (Ubi-K48, K48 WT only) had no effect on ECH1 ubiquitination (Figure 3F), indicating that TRAF3 ubiquitinates ECH1 through K63-linked ubiquitin chains.

Molecular docking showed that Lys214 and Lys276 in the C-terminal of ECH1 were located at the interaction interface between ECH1 and TRAF3, with interatomic distances of 1.9 and 4.4 Å, respectively. These 2 residues were found to be conserved among different species (Figure 3G) (29), suggesting potential ubiquitination of the residues by TRAF3. Therefore, we mutated these 2 residues (Lys to Arg) individually and found that K214R almost abolished ECH1 ubiquitination by TRAF3, whereas K276R did not (Figure 3H and Supplemental Figure 3D). Moreover, in vitro ubiquitination assays using purified proteins showed that TRAF3 ubiquitinated WT ECH1 in a K63-linked manner, whereas the ECH1-K214R mutant was not ubiquitinated (Figure 3I). Importantly, as with other K63-linked ubiquitinations mediated by the TRAF family (30), we found that overexpression of TRAF3 had no effect on the expression or stability of the ECH1 protein in GBM cells (Supplemental Figure 3E). Together, our findings indicate that TRAF3 preferentially ubiquitinated ECH1 through K63-linked ubiquitin chains at Lys214.

### TRAF3-mediated ubiquitination of ECH1 inhibits its interaction with TOMM20 and mitochondrial translocation.

**Figure 4:**
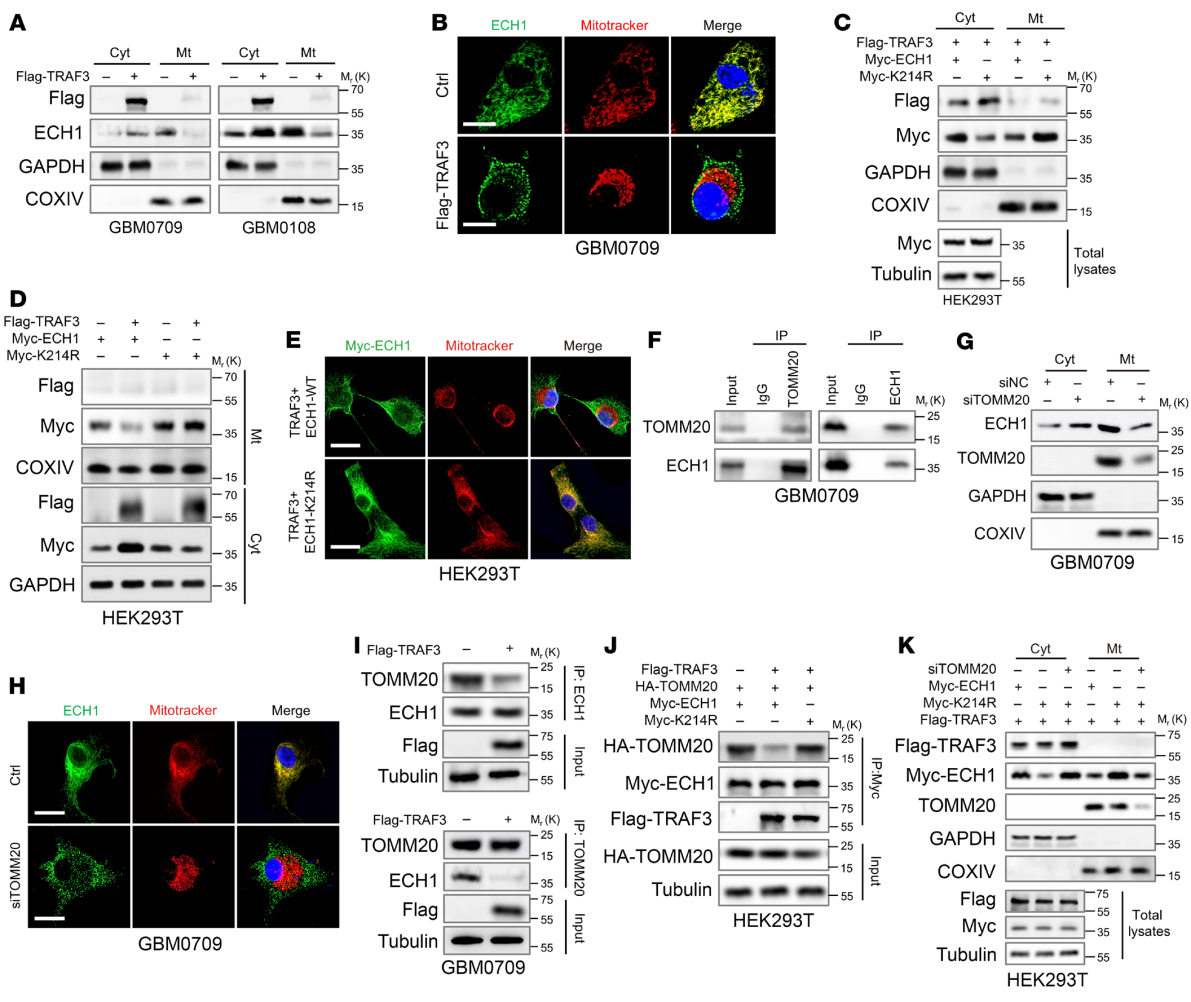
TRAF3-mediated ubiquitination of ECH1 inhibits its mitochondrial translocation. (**A**) GBM0709 and GBM0108 cells were transfected with Flag-TRAF3 or a control plasmid, and the cytosolic (Cyt) and mitochondrial (Mt) protein fractions were isolated and then were subjected to immunostaining using the indicated antibodies. (**B**) IF assays demonstrate the subcellular location of ECH1 in GBM0709 cells transfected with Flag-TRAF3. Scale bars: 20 μm. (**C**) The cytosolic and mitochondrial levels of ECH1-WT or ECH1-K214R in HEK293T cells expressing Flag-TRAF3 were evaluated by immunoblotting. (**D**) HEK293T cells were transfected with TRAF3 and ECH1-WT or ECH1-K214R, and the cytosolic and mitochondrial levels of ECH1 were evaluated by immunoblotting. (**E**) HEK293T cells were transfected with TRAF3 and ECH1-WT or ECH1-K214R, and the subcellular location of ECH1 was detected by immunostaining. Scale bars: 20 μm. (**F**) GBM0709 cell lysates were immunoprecipitated with an antibody against ECH1 or TOMM20, and the resultant immunoprecipitates were subjected to immunoblotting. (**G**) GBM0709 cells were transfected with TOMM20 siRNA (siTOMM20) or normal control siRNA (siNC), and the cytosolic and mitochondrial protein fractions were subsequently analyzed by immunoblotting. (**H**) GBM0709 cells were transfected with TOMM20 siRNA, and the subcellular location of ECH1 were analyzed by IF. Scale bars: 20 μm. (**I**) GBM0709 cells were transfected with TRAF3, and cell lysates were incubated with an antibody against ECH1 or TOMM20 followed by immunoblotting. (**J**) HEK293T cells were transfected with Flag-TRAF3, HA-TOMM20, and ECH1-WT or ECH1-K214R, and cell lysates were immunoprecipitated with an anti–Myc-tag antibody followed by immunoblotting. (**K**) HEK293T cells were transfected with Flag-TRAF3, TOMM20 siRNA, and ECH1-WT or ECH1-K214R, and the cytosolic and mitochondrial protein fractions were isolated and then analyzed by immunoblotting using the indicated antibody.

Although K48-linked ubiquitination typically results in protein degradation, K63-linked ubiquitination is primarily involved in the regulation of protein function and subcellular localization (30). Given that ECH1 is predominantly localized in mitochondria and involved in the metabolism of UFAs, we hypothesized that TRAF3-mediated K63 ubiquitination of ECH1 may regulate its mitochondrial translocation. In GBM0709 and GBM0108 cells, the majority of the ECH1 proteins were observed to localize to mitochondria (Figure 4A and Supplemental Figure 4A). TRAF3 overexpression resulted in an increase in the fraction of cytoplasmic ECH1 and a concomitant decrease in mitochondrial ECH1 (Figure 4A and Supplemental Figure 4A). These results were confirmed by immunostaining of GBM0709 and HEK293T cells (Figure 4B and Supplemental Figure 4B). Notably, overexpression of TRAF3 did not significantly impede the mitochondrial translocation of ECH1-K214R (Figure 4, C–E), indicating that TRAF3 inhibited ECH1 mitochondrial translocation in a K63-linked ubiquitination–dependent manner.

The translocase of the outer mitochondrial membrane (TOM) complex plays a vital role in transporting mitochondrial enzymes from the cytoplasm to the inner space of mitochondria. This complex is primarily composed of TOMM20, TOMM22, and TOMM40, with TOMM20 serving as a guide for the interaction between transported proteins and the TOM complex by binding to the mitochondrial leader sequences located in the N-terminus of the target proteins (31, 32). The BioGRID protein interaction database indicated a potential interaction of ECH1 with TOMM20 and TOMM22 (Supplemental Figure 4C). We subsequently confirmed the interaction between ECH1 and TOMM20 by reciprocal immunoprecipitation and immunofluorescence (IF) assays (Figure 4F and Supplemental Figure 4, D–F). Furthermore, depletion of TOMM20 substantially impeded the translocation of ECH1 into the mitochondria (Figure 4, G and H), indicating a requirement of TOMM20 for ECH1 mitochondrial translocation. We next investigated the effect of TRAF3-mediated K63 ubiquitination of ECH1 on its interaction with TOMM20. We found that TRAF3 overexpression notably disrupted the interaction between ECH1 and TOMM20 (Figure 4I). Importantly, while TRAF3 overexpression effectively impaired the association of TOMM20 and WT ECH1, it had no effect on the interaction between TOMM20 and ECH1-K214R (Figure 4J). Accordingly, while TRAF3 overexpression did not interrupt the translocation of ECH1-K214R into mitochondria (Figure 4D), concurrent TOMM20 depletion substantially impeded the mitochondrial translocation of ECH1-K214R (Figure 4K). Collectively, these results indicate that TRAF3-mediated K63 ubiquitination of ECH1 inhibited its interaction with TOMM20, thereby disrupting its mitochondrial translocation.

### Depletion of ECH1 promotes the accumulation of PUFAs and lipid peroxidation.

**Figure 5:**
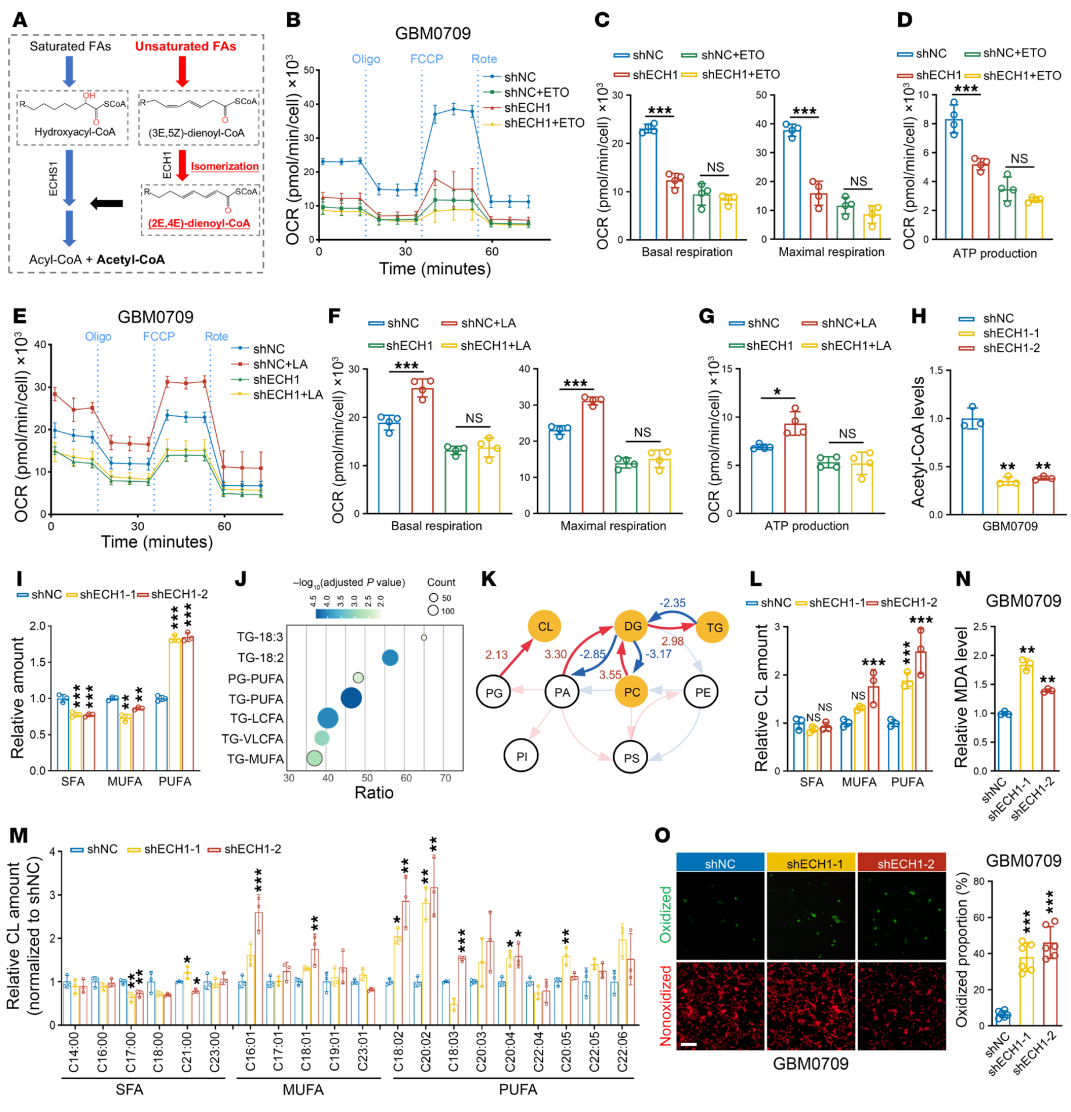
Depletion of ECH1 induces accumulation of PUFAs and lipid peroxidation. (**A**) ECH1 catalyzes the isomerization of a 3-trans, 5-cis dienoyl-CoA substrate to a 2-trans, 4-trans dienoyl-CoA product, enabling UFAs oxidation. (**B**) OCR time series in shECH1 GBM0709 cells treated or not with ETO by Seahorse assay. (**C** and **D**) Quantification of basal and maximum respiration (**C**) and ATP production (**D**) in GBM0709 cells. (**E**) OCR time series in shECH1 GBM0709 cells treated or not with BSA-conjugated linoleic acid (LA). (**F** and **G**) Quantification of basal and maximum respiration (**F**) and ATP production (**G**) in GBM0709 cells. Data are expressed as the mean ± SD of 4 independent assays (**B**–**G**). (**H**) Acetyl-CoA levels in GBM0709 cells expressing shECH1 compared with control shRNA (*n* = 3). (**I**) Lipidomics analysis shows the relative content of SFAs, MUFAs, and PUFAs in GBM0709 cells expressing shECH1 compared with control shRNA (*n* = 3). (**J**) Enrichment analysis of upregulated lipid species in GBM0709 cells expressing shECH1-1 compared with control shRNA. (**K**) Biosynthetic analysis of lipid species in GBM0709 cells expressing shECH1-1 compared with control shRNA. (**L**) Relative content of SFAs, MUFAs, and PUFAs in CL in GBM0709 cells expressing ECH1 shRNAs compared with control (*n* = 3). (**M**) Analysis of the specific FA branches in CL in GBM0709 cells expressing shECH1 compared with control shRNAs (*n* = 3). (**N**) MDA levels were detected in GBM0709 cells expressing shECH1 (*n* = 3). (**O**) BODIPY 581/591 staining of GBM0709 cells expressing shECH1. Scale bar: 100 μm. The proportion of oxidized cells was calculated (*n* = 6). Statistical significance was determined by 1-way ANOVA with Tukey’s post hoc test (**C**, **D**, **F**–**I**, and **L**–**O**). Oligo, oligonucleotide; Rote, rotenone. Data are expressed as the mean ± SD. *P < 0.05, **P < 0.01, and ***P < 0.001.

Unlike SFAs, which can directly undergo mitochondrial β-oxidation, UFAs require specific auxiliary enzymes to facilitate their complete oxidation (25, 33). ECH1 is one such enzyme that catalyzes the *cis-trans* isomerization of UFAs with double bonds at odd-numbered positions along the carbon chain (Figure 5A) and has been proposed to play an important role in facilitating complete UFA metabolism (25, 33). Therefore, we investigated the role of ECH1 in mitochondrial oxidative metabolism of GBM cells. The expression level of *ECH1* was not significantly different in different grades of gliomas (Supplemental Figure 5A). As anticipated, we found that depletion of ECH1 significantly decreased both the basal and maximal respiration capacity, as well as ATP production, as determined by oxygen consumption rate (OCR) analysis in GBM0709 and GBM0108 cells (Figure 5, B–D, and Supplemental Figure 5, B–E). However, upon the addition of etomoxir (ETO), a CPT1 inhibitor that blocks the transport of long-chain FAs into mitochondria for FAO (34), ECH1 depletion did not further decrease the basal and maximal respiration capacity or ATP production in GBM cells (Figure 5, B–D, and Supplemental Figure 5, C–E). Similarly, although the supply of the PUFA LA-BSA stimulated mitochondrial respiration through FAO in GBM cells, we observed no significant stimulation following ECH1 depletion (Figure 5, E–G, and Supplemental Figure 5, F–H). Moreover, the levels of acetyl-CoA, the end-product of FAO, were markedly reduced following ECH1 depletion (Figure 5H and Supplemental Figure 5I). Thus, these results demonstrate that ECH1 is a pivotal regulator of FAO in GBM cells.

To gain insights into the metabolic alterations underlying the effect of ECH1 on FAO, we conducted lipidomics analysis following ECH1 silencing (Supplemental Table 4). The results showed that depletion of ECH1 significantly promoted the accumulation of PUFAs, while decreasing the content of SFAs and MUFAs (Figure 5I). Notably, ECH1 depletion resulted in a significant increase in several specific lipid species, including triglycerides (TGs), phosphatidylglycerol (PG), and glycerolipids (GLSs) (Figure 5J and Supplemental Figure 5J). A comprehensive analysis of biosynthetic pathways based on lipidomics data (35), conducted using BioPAN, further revealed an increase in the synthesis of cardiolipin (CL) and TGs (Figure 5K and Supplemental Figure 5K). Consistently, we observed that the content of PUFAs was increased in CL and TGs upon ECH1 silencing (Figure 5L and Supplemental Figure 5L). Specifically, the PUFA branches including C18:2, C20:2, and C18:3 were significantly increased in CL after ECH1 depletion (Figure 5M), indicating a preference of ECH1 in the metabolism of PUFAs in CL.

The accumulation of unmetabolized PUFAs usually triggers peroxidation (4). Moreover, CL, which is primarily composed of UFAs, is a characteristic lipid component of the mitochondrial membrane and is prone to peroxidation and oxidative damage, leading to mitochondrial dysfunction and collapse (36, 37). Therefore, we proceeded to investigate the effect of ECH1 depletion on lipid peroxidation. As anticipated, depletion of ECH1 in GBM0709 and GBM0108 cells significantly upregulated the levels of malondialdehyde (MDA), a primary product of lipid peroxidation (Figure 5N and Supplemental Figure 5M). Moreover, BODIPY 581/591 staining also revealed a higher percentage of lipid peroxidation in GBM cells following ECH1 depletion (Figure 5O and Supplemental Figure 5N). Together, these results demonstrate that ECH1 depletion impeded the oxidation of PUFAs and stimulated lipid peroxidation in glioma cells.

### ECH1 depletion triggers ROS-related mitochondrial damage and inhibits GBM tumorigenesis.

**Figure 6:**
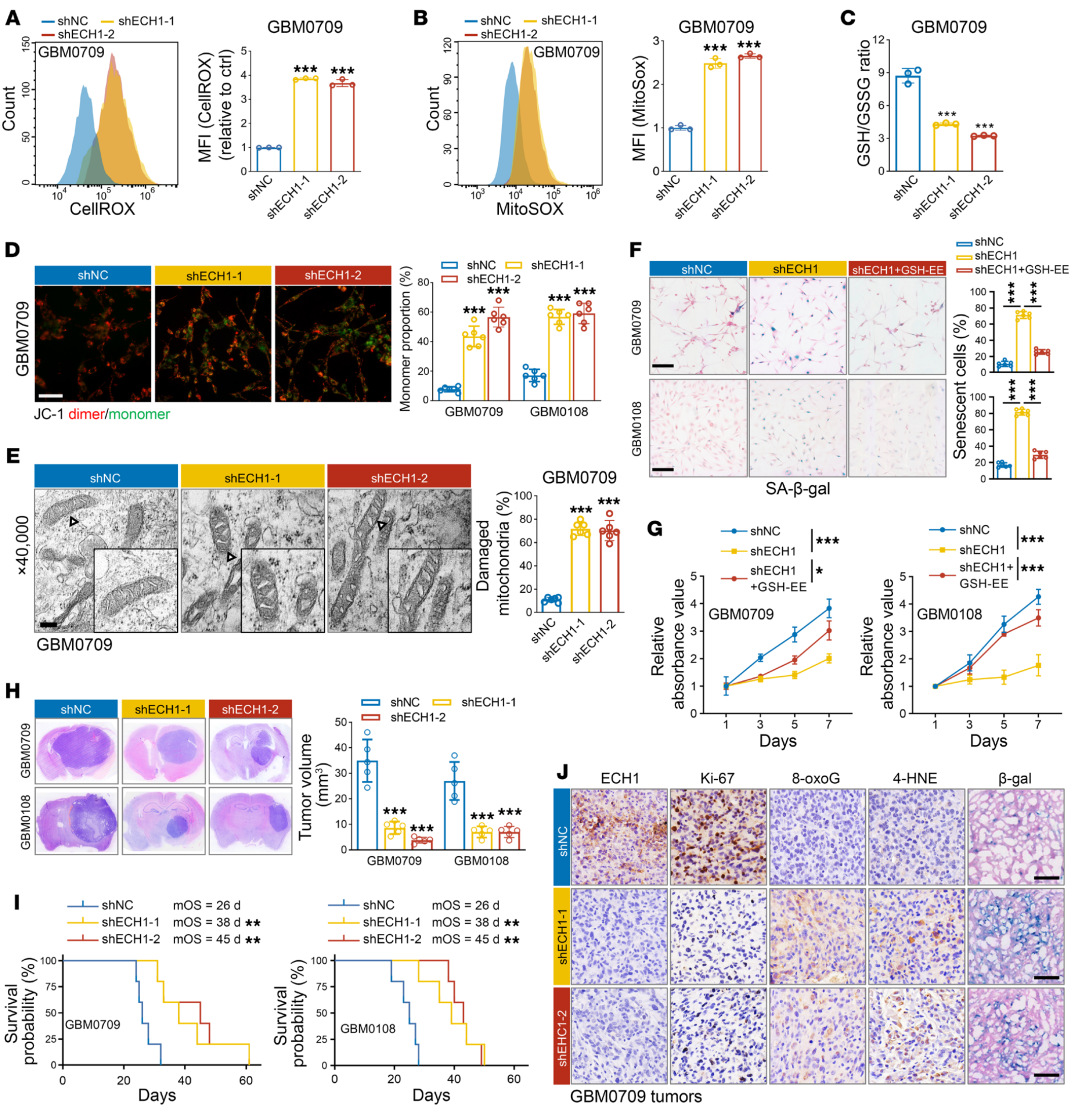
ECH1 depletion triggers ROS-related mitochondrial damage and inhibits GBM tumorigenesis. (**A** and **B**) CellROX (**A**) and MitoSOX (**B**) levels of GBM0709 cells stably expressing *ECH1* shRNAs were analyzed by flow cytometry. Representative flow cytometry plots and MFI quantification (mean ± SD, *n* = 3 independent experiments) are shown. ***P < 0.001. (**C**) GSH/GSSG ratios were evaluated in GBM0709 cells stably expressing *ECH1* shRNAs. Data are expressed as the mean ± SD of 3 independent experiments. ***P < 0.001. (**D**) JC-1 staining in GBM0709 cells stably expressing *ECH1* shRNAs. Representative images are shown. Scale bar: 100 μm. The proportions of JC-1 monomer (green) in each group were statistically analyzed (mean ± SD, *n* = 6 randomly selected microscope fields). ***P < 0.001. (**E**) TEM images of mitochondria in GBM0709 cells stably expressing *ECH1* shRNAs. Scale bar: 500 nm (original magnification, ×40,000; enlarged magnification, ×80,000). The proportion of damaged mitochondria was statistically analyzed (mean ± SD, *n* = 6 randomly selected microscope fields). ***P < 0.001. (**F**) GBM0709 and GBM0108 cells stably expressing *ECH1* shRNA were treated or not with GSH, and cells were then stained for β-gal. Representative images are shown. Scale bars: 100 μm. The percentage of β-gal^+^ cells was determined (mean ± SD, *n* = 6 randomly selected microscope fields). ***P < 0.001. (**G**) GBM0709 and GBM0108 cells stably expressing *ECH1* shRNA were treated or not with GSH, and cell viability was evaluated by CCK8. Absorbance values were normalized to the control (mean ± SD, *n* = 4 independent assays). *P < 0.05 and ***P < 0.001. (**H**) GBM0709 and GBM0108 cells stably expressing *ECH1* shRNAs were i.c. injected into nude mice. H&E-stained sections show representative tumor xenografts. Tumor volumes were calculated (mean ± SD, *n* = 5 mice for each group). ***P < 0.001. (**I**) Survival of GBM tumor–bearing mice (*n* = 5 mice for each group, Kaplan-Meier model). **P < 0.01. (**J**) Mouse brain tissues derived from GBM0709 cells were stained for ECH1, Ki-67, 8-oxoG, 4-HNE, and SA–β-gal, respectively. Scale bars: 200 μm. Statistical analysis was performed using 1-way ANOVA with Tukey’s post hoc test (**A**–**H**) or log-rank test (**I**).

Because UFAs are susceptible to oxidative damage, we hypothesized that accumulation of unmetabolized UFAs induced by ECH1 depletion may lead to mitochondrial failure. Consistent with this hypothesis, quantification of ROS production demonstrated that ECH1 depletion in GBM cells resulted in increased levels of cellular and mitochondrial ROS (Figure 6, A and B, and Supplemental Figure 6, A and B), along with a decreased GSH/GSSG ratio (Figure 6C and Supplemental Figure 6C). Accordingly, ECH1 depletion decreased mitochondrial membrane potential, as determined by JC-1 staining (Figure 6D and Supplemental Figure 6D). Furthermore, TEM images revealed that ECH1 depletion led to damaged mitochondria with darker matrices and swollen cristae (Figure 6E). Thus, these results indicate that ECH1 depletion promoted ROS-induced mitochondrial damage.

We next explored the role of ECH1 in cell growth and GBM tumorigenesis. As with TRAF3 overexpression, depletion of ECH1 reduced the levels of clusterin, p53, and p21 in GBM cells, and these effects were reversed by GSH-EE (Supplemental Figure 6F). Accordingly, ECH1 depletion significantly induced cellular senescence and inhibited cell viability and colony formation of GBM cells, which were all reversed by treatment with GSH-EE (Figure 6, F and G, and Supplemental Figure 6E). In an orthotopic xenograft GBM model, ECH1 depletion in GBM0709 and GBM0108 cells resulted in significant inhibition of tumor growth (Figure 6H) and a considerable prolongation of the survival of GBM tumor–bearing mice (Figure 6I). In the mouse GBM tissues, ECH1 depletion was associated with a notable decline in Ki-67 expression compared with the control group, accompanied by elevated levels of 8-oxoG, 4-hydroxynonenal (4-HNE), and β-gal staining, which are indicative of oxidative DNA, lipid damage, and cellular senescence, respectively (Figure 6J and Supplemental Figure 6G). Taken together, these findings provide compelling evidence that ECH1 depletion triggered ROS-related mitochondrial damage and inhibited cell growth and GBM tumorigenesis.

### TRAF3 impedes FAO and induces lipid peroxidation through ubiquitination of ECH1.

**Figure 7:**
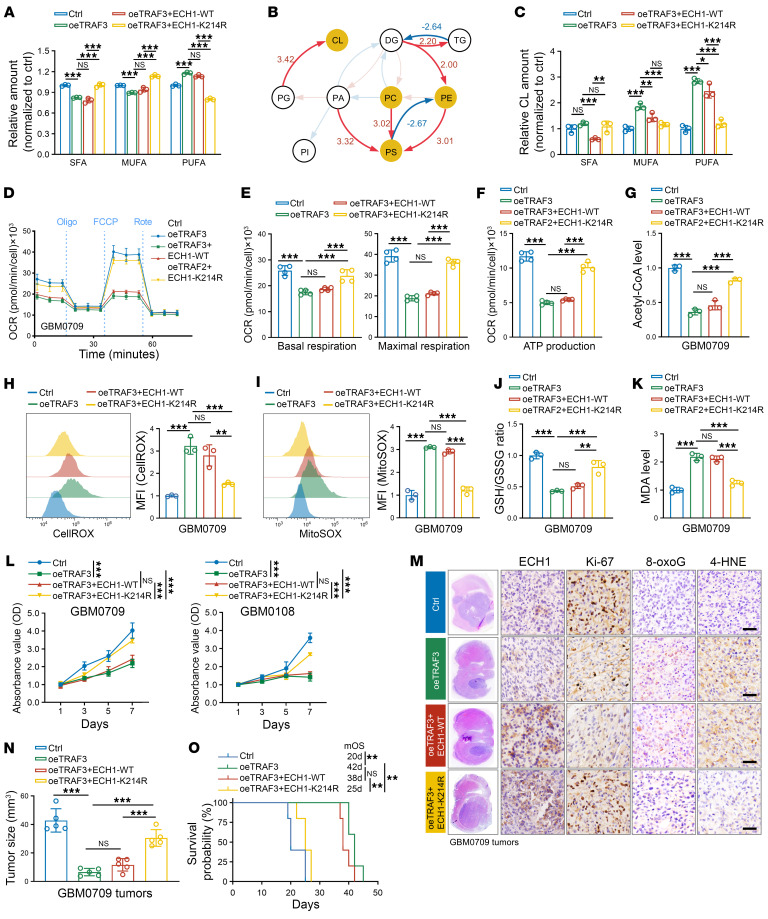
TRAF3 impedes FAO and induces lipid peroxidation in GBM cells through ubiquitination of ECH1. (**A**) Relative content of SFAs, MUFAs, and PUFAs in GBM0709 cells expressing TRAF3, TRAF3+ECH1-WT, or TRAF3+ECH1-K214R (*n* = 3). (**B**) Biosynthetic analysis of lipid species in GBM0709 cells expressing TRAF3 compared with the control group. (**C**) Relative content of SFAs, MUFAs, and PUFAs in CL in GBM0709 cells expressing TRAF3, TRAF3+ECH1-WT, or TRAF3+ECH1-K214R (*n* = 3). (**D**) OCR time series in GBM0709 cells expressing TRAF3, TRAF3+ECH1-WT, or TRAF3+ECH1-K214R. (**E** and **F**) Quantification of basal respiration and maximum respiration (**E**) and ATP production (**F**) in GBM0709 cells. (**G**) Quantification of acetyl-CoA levels in GBM0709 cells. In **D**–**G**, Data are expressed as the mean ± SD of 3 or 4 independent experiments. (**H** and **I**) Flow cytometric analysis of CellROX (**H**) and MitoSOX (**I**) staining in GBM0709 cells expressing TRAF3, TRAF3+ECH1- WT, or TRAF3+ECH1-K214R (*n* = 3). (**J**) GSH/GSSG ratios in GBM0709 cells expressing TRAF3, TRAF3+ECH1-WT, or TRAF3+ECH1-K214R (*n* = 3). (**K**) MDA levels in GBM0709 cells expressing TRAF3, TRAF3+ECH1-WT, or TRAF3+ECH1-K214R (*n* = 3). (**L**) Cell viability of GBM0709 and GBM0108 cells stably expressing TRAF3, TRAF3+ECH1-WT, or TRAF3+ECH1-K214R was evaluated by CCK8, normalized to the control (*n* = 4). (**M** and **N**) GBM0709 cells stably expressing TRAF3, TRAF3+ECH1-WT, or TRAF3+ECH1-K214R were i.c. injected into nude mice. H&E-stained sections show representative tumor xenografts (**M**). Mouse tumor tissues were stained for ECH1, Ki-67, 8-oxoG, and 4-HNE, respectively (**M**). Scale bars: 100 μm. Tumor volumes were calculated (*n* = 5) (**N**). (**O**) Survival of GBM0709 GBM tumor–bearing mice (*n* = 5 mice, Kaplan-Meier model). Statistical significance was determined by 1-way ANOVA with Tukey’s post hoc test (**A**, **C**, **E**–**L**, and **N**) or log-rank test (**O**). Data represent the mean ± SD. *P < 0.05, **P < 0.01, and ***P < 0.001.

We next determined whether TRAF3 regulates FAO and lipid peroxidation through the ubiquitination of ECH1. To this end, we first conducted lipidomics analysis to examine the changes in lipid metabolism following TRAF3 overexpression. The results demonstrated that TRAF3 overexpression led to a reduction in the levels of SFAs and MUFAs and an increase in the accumulation of PUFAs (Figure 7A and Supplemental Table 5), which was consistent with the effect observed with ECH1 knockdown. Additionally, we detected an increase in the levels of CL following TRAF3 overexpression (Figure 7B), as well as an elevated proportion of PUFAs and MUFAs in the CL branch (Figure 7C). Notably, although overexpression of WT ECH1 had no effect or a modest reversal of the effects of TRAF3 overexpression on PUFA levels, overexpression of ECH1-K214R substantially decreased the proportion of PUFAs caused by TRAF3 overexpression (Figure 7, A and C).

We proceeded to assess the effect of TAFR3-mediated ECH1 ubiquitination on mitochondrial respiration. Our findings revealed that TRAF3 overexpression markedly reduced mitochondrial respiration (Figure 7, D–F, and Supplemental Figure 7, A–C), similar to the effect observed with ECH1 knockdown. Further analysis revealed that the reconstitution of WT ECH1 expression in GBM cells only marginally rescued the inhibitory effect of TRAF3 overexpression on the basal and maximal respiration capacity as well as on ATP production (Figure 7, D–F, and Supplemental Figure 7, A–C). However, ECH1-K214R almost completely restored the oxidation capacity of GBM cells that was repressed by TRAF3 overexpression (Figure 7, D–F, and Supplemental Figure 7, A–C). Notably, despite the established role of TRAF3 as an inhibitor of the NF-κB pathway in glioma, restoration of the alternative NF-κB pathway by expression of MAP3K14 did not rescue the effect of TRAF3 overexpression on the oxidation capacity of GBM cells (Supplemental Figure 7, D–G). This indicates that TRAF3 regulated glioma metabolism independently of the NF-κB pathway. Consistently, compared with WT ECH1, overexpression of ECH1-K214R was significantly more potent in upregulating the levels of acetyl-CoA that were suppressed by TRAF3 overexpression (Figure 7G and Supplemental Figure 7H). Moreover, while WT ECH1 only slightly reversed the effect of TRAF3 overexpression on cellular and mitochondrial ROS levels, GSH/GSSG ratios, and MDA levels, ECH1-K214R significantly reversed those effects induced by TRAF3 overexpression (Figure 7, H–K, and Supplemental Figure 7, I–L). In accordance with these findings, overexpression of ECH1-K214R, but not WT ECH1, substantially rescued GBM cells from the growth arrest caused by TRAF3 overexpression (Figure 7L and Supplemental Figure 7M).

In an orthotopic GBM model, we observed that reconstituted expression of ECH1-K214R in GBM cells with TRAF3 overexpression was more potent in promoting GBM growth compared with WT ECH1 and, accordingly, markedly shortened the survival of GBM0709 GBM tumor–bearing mice (medium survival of 38 days for oeTRAF3 + ECH1-WT mice vs. 25 days for oeTRAF3 + ECH1-K214R mice) (Figure 7, M–O). In mouse GBM tissues, ECH1-K214R, but not WT ECH1, tissues had significantly elevated levels of Ki-67, with concomitantly reduced 8-oxoG and 4-HNE levels, which were affected by TRAF3 overexpression (Figure 7M). Collectively, these results demonstrate that TRAF3 inhibited FAO and promoted lipid peroxidation through the ubiquitination of ECH1.

### TRAF3 overexpression sensitizes GBM to ferroptosis and anti–PD-L1 therapy.

**Figure 8:**
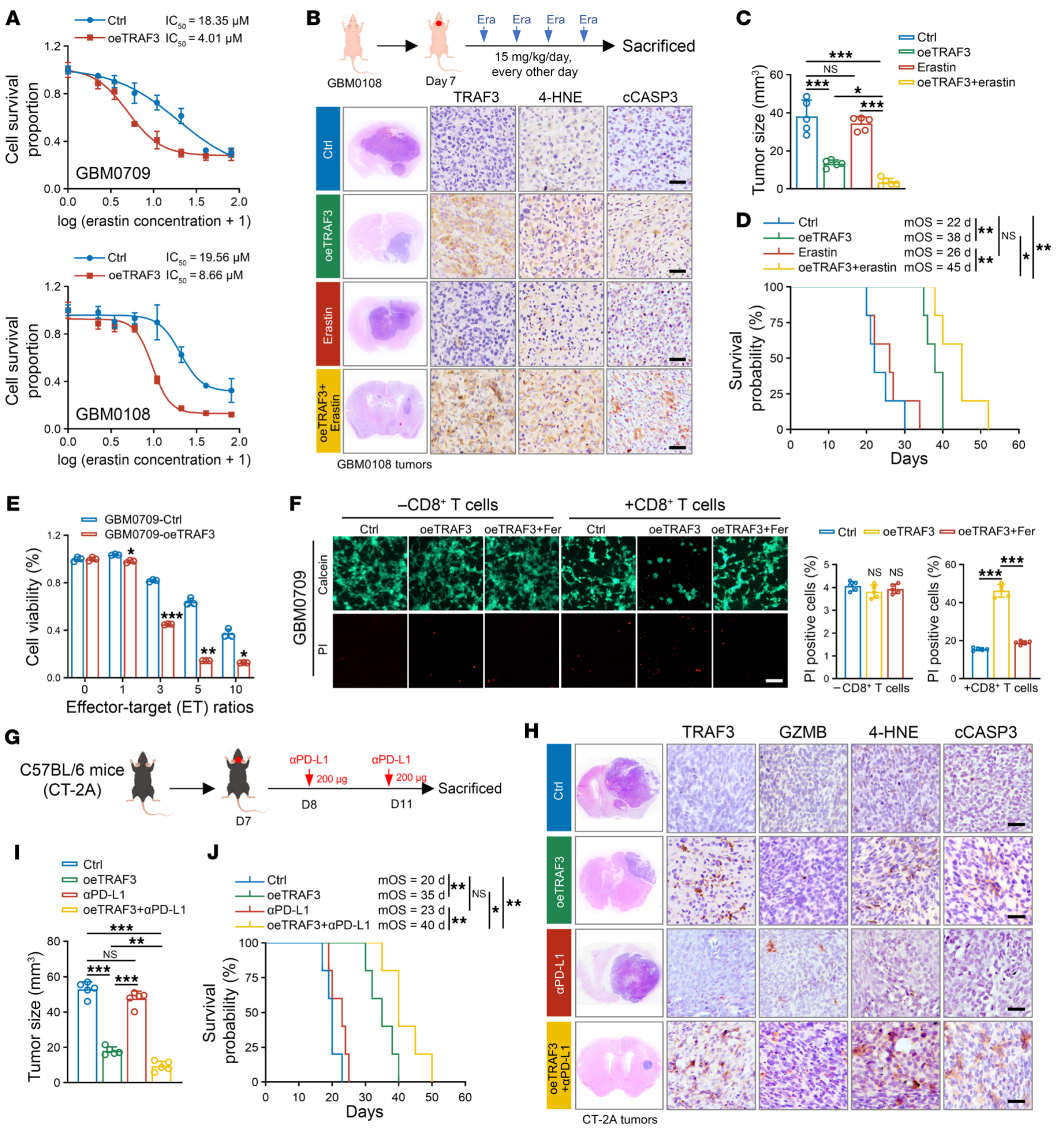
Overexpression of TRAF3 sensitizes GBM to erastin and anti–PD-L1 therapy. (**A**) GBM0709 and GBM0108 cells expressing TRAF3 were treated with different concentration of erastin for 72 hours, and IC_50_ values for each cell line were calculated (mean ± SD, *n* = 4 independent experiments). (**B** and **C**) GBM0108 cells (5 × 10^5^ cells/mouse) stably expressing TRAF3 were i.c. injected into nude mice. Mice were then i.p. injected with erastin (15 mg/kg/d) every other day. H&E-stained sections show representative tumor xenografts (**B**). The mouse tumor tissues were stained with TRAF3, 4-HNE, and cleaved caspase 3 (cCASP3), respectively (**B**). Scale bars: 100 μm. Tumor volumes were calculated (**C**) (mean ± SD, *n* = 5 mice for each group). *P < 0.05 and ***P < 0.001. (**D**) Survival of the mice (*n* = 5 mice for each group, Kaplan-Meier model). *P < 0.05 and **P < 0.01. (**E**) GBM0709 cells expressing TRAF3 were cocultured with activated CD8^+^ T cells for 48 hours with different E/T ratios (from 1:1 to 10:1), and cell viability was evaluated (mean ± SD, *n* = 3 independent experiments). *P < 0.05, **P < 0.01, and ***P < 0.001. (**F**) GBM0709 cells expressing TRAF3 were treated with ferrostatin (Fer) or DMSO and then cocultured with activated CD8^+^ T cells for 48 hours (E/T ratio = 3:1). The surviving and dead cells were stained by calcein-AM/PI. Representative images are shown. Scale bar: 100 μm. The percentage of PI^+^ cells was counted (mean ± SD, *n* = 5 randomly selected microscope fields). ***P < 0.001. (**G**–**I**) CT-2A cells stably expressing *TRAF3* were i.c. injected into C57BL/6 mice. Mice were then i.p. injected twice with anti–PD-L1 mAb (200 μg/mouse/d) (**G**). H&E-stained sections show representative tumor xenografts. Tumor tissues were stained for TRAF3, GZMB, 4-HNE, and cleaved caspase 3, respectively (**H**). Scale bars: 100 μm. Tumor volumes were calculated (**I**) (mean ± SD, *n* = 5 mice for each group). **P < 0.01, ***P < 0.001. (**J**) Survival of the CT-2A GBM tumor–bearing mice (*n* = 5 mice for each group, Kaplan-Meier model). *P < 0.05 and **P < 0.01. Statistical analysis was performed using 1-way ANOVA with Tukey’s post hoc test (**C**, **E**, **F**, and **I**) or a log-rank test (**D** and **J**).

Accumulation of PUFAs and lipid peroxidation are hallmarks of ferroptosis (38). Therefore, we next investigated the role of TRAF3 in GBM cell ferroptosis. As anticipated, TRAF3 overexpression substantially enhanced erastin-induced ferroptosis in GBM0709 cells (IC_50_ = 18.35 μM in the control group vs. 4.01 μM in the oeTRAF3 group), GBM0108 cells (19.56 μM vs. 8.66 μM), and U87MG cells (7.27 μM vs. 3.76 μM) (Figure 8A and Supplemental Figure 8A). However, treatment with ferrostatin, a ferroptosis inhibitor, did not rescue the growth inhibition effect caused by TRAF3 overexpression (Supplemental Figure 8B), indicating that TRAF3 sensitized GBM cells to ferroptosis rather than directly inducing it. Consistently, in an orthotopic GBM model using erastin-resistant GBM0108 cells, we found that erastin treatment alone did not significantly promote lipid peroxidation ot cell death in the tumors and that GBM tumor–bearing mice did not benefit from erastin treatment (22 days in control mice vs. 26 days in erastin-treated mice) (Figure 8, B–D). However, in the oeTRAF3 group, concomitant erastin treatment substantially induced lipid peroxidation and cell death in the tumors. This combination significantly inhibited tumor growth and prolonged the overall survival of GBM tumor–bearing mice (22 days for control mice vs. 45 days for oeTRAF3 + erastin mice) (Figure 8, B–D).

It has been reported that CD8^+^ T cells mediate tumor cell killing primarily through ferroptosis and that blocking T cell–induced tumor cell ferroptosis leads to resistance to immune checkpoint inhibitor (ICI) therapy (39, 40). Therefore, we investigated the effect of TRAF3 overexpression on CD8^+^ T cell–mediated cell cytotoxicity. To this end, GBM cells were cocultured with activated CD8^+^ T cells at varying effector/target (E/T) ratios. As we expected, although cell viability decreased with increasing E/T ratios, GBM cells expressing TRAF3 were more susceptible to CD8^+^ T cell–mediated killing in comparison with control group cells (Figure 8E and Supplemental Figure 8C). Notably, the proportion of GZMB^+^ cells in the CD8^+^ T cell population was not obviously altered between the control and oeTRAF3 groups (Supplemental Figure 8, D and E), suggesting that TRAF3 overexpression in GBM cells did not affect the cytotoxicity of CD8^+^ T cells, but instead affected the susceptibility of tumor cells to T cells. Further investigation using propidium iodide/calcein-acetoxymethyl (PI/calcein-AM) staining substantiated that ferrostatin treatment markedly impaired the killing of GBM cells by CD8^+^ T cells that had been induced by TRAF3 overexpression (Figure 8F and Supplemental Figure 8F). These findings indicate that TRAF3 rendered GBM cells susceptible to T cell killing by enhancing ferroptosis sensitivity.

To investigate the effect of TRAF3 overexpression on anti–PD-L1 immunotherapy, we established an orthotopic xenograft GBM model using mouse CT-2A and GL261 GBM cells, respectively (Figure 8G and Supplemental Figure 8,G and H). Consistent with the in vitro assays, anti–PD-L1 treatment alone activated the antitumor CD8^+^ T cell response, yet did not significantly induce tumor cell death or inhibit tumor growth (Figure 8, H–J, and Supplemental Figure 8, H–L). In contrast, in the oeTRAF3 group, concomitant anti–PD-L1 treatment not only increased the population of cytolytic CD8^+^ T cells but also significantly induced lipid peroxidation and cell death in mouse tumors (Figure 8, H–J and Supplemental Figure 8, H–L). As a result, the combination of PD-L1 blockade and TRAF3 overexpression significantly reduced the tumor burden and prolonged the survival of CT-2A GBM tumor–bearing mice (23 days for PD-L1 mAb mice vs. 40 days for PD-L1 mAb + oeTRAF3 mice) (Figure 8, H–J). Similar outcomes were also observed for GL261 GBM tumor–bearing mice (20.5 days for PD-L1 mAb mice vs. 45.5 days for PD-L1 mAb + oeTRAF3 mice) (Supplemental Figure 8M). Notably, the combined therapeutic effect was eliminated when the GL261 GBM tumor–bearing mice were depleted of CD8^+^ T cells (45.5 days in oeTRAF3 + PD-L1 mAb mice vs. 37 days in oeTRAF3 + PD-L1 mAb + CD8α mAb mice) (Supplemental Figure 8M). Taken together, these findings provide compelling evidence that TRAF3-induced lipid peroxidation enhanced the susceptibility of GBM cells to ferroptosis-inducing agents and anti–PD-L1 immunotherapy, suggesting a promising therapeutic strategy for GBM.

## Discussion

Dysregulation of lipid metabolism is a hallmark of cancer. Tumor cells utilize FAs to provide the necessary energy and building blocks for sustained tumor growth and to prevent potential lipotoxicity caused by the accumulation of unmetabolized UFAs, although the underlying mechanism remains largely unknown. In the present study, we demonstrate that the loss of TRAF3 in GBM activated ECH1-mediated metabolism of PUFAs, thereby inhibiting lipid peroxidation and promoting tumor growth. Overexpression of TRAF3 in GBM cells induced the accumulation of unmetabolized PUFAs and peroxidation mitochondrial damage, leading to increased sensitivity of GBM to ferroptosis and anti–PD-L1 immunotherapy, which indicates a promising strategy for GBM treatment.

Compared with other tumors, GBM has distinctive characteristics in lipid metabolism. Dysregulated expression of genes associated with lipid metabolism is frequent in GBM. These include genes for enzymes responsible for FA synthesis (12), FAO (41), lipid transporters (42), and regulatory proteins involved in lipid homeostasis (7). As a result, GBM cells exhibit unique lipid profiles compared with normal glial cells and other tumor types (43). Such altered lipid profiles can affect a number of crucial cellular processes, including cell signaling, membrane structure, and overall cellular function. GBM cells display an increased reliance on FAO as a source of energy. This heightened FAO activity enables GBM cells to meet their increased energy demands and survive in nutrient-depleted conditions, such as limited glucose and oxygen availability, which are commonly observed in the GBM microenvironment (5, 44). Furthermore, GBM tumors frequently exhibit augmented accumulation of lipid droplets, which serve as intracellular lipid storage sites (45). These lipid droplets function as energy reservoirs and building blocks for membrane synthesis, thereby facilitating the rapid proliferation and invasive behavior of GBM cells (45).

While the majority of research focuses on the energy production aspect of enhanced FAO in cancer, it is important to recognize that FAO plays a multitude of roles beyond ATP production. These include cataplerotic reactions that provide substrates for amino acids, nucleotide synthesis, and improved redox potential (41, 46–48). GBM cells have been observed to upregulate the FAO pathway as a protective mechanism against lipid peroxidation, particularly of UFAs, which are susceptible to oxidative damage due to the presence of double bonds (38, 43). By enhancing FAO, GBM cells can minimize the accumulation of UFAs and reduce lipid peroxidation, thereby promoting the survival and proliferation of these GBM cells (38). The dependency of malignant cells on FAO may represent a distinctive metabolic vulnerability. For instance, the inhibition of medium-chain acyl-CoA dehydrogenase (MCAD) did not elicit cytotoxic or antiproliferative effects in normal glial cells, but decreased MCAD function in GBM cells resulted in a toxic accumulation of lipids, which triggered mitochondrial failure (38). In the present study, we made an intriguing observation that TRAF3 was specifically associated with favorable survival outcomes in glioma cohorts compared with other types of tumors. This finding suggests that the catabolism pattern of PUFAs under the control of the TRAF3/ECH1 axis may be specific to glioma, potentially because of the vulnerability of GBM to lipid peroxidation. The dysregulated lipid metabolism in GBM, along with the specific role of TRAF3 in modulating PUFA catabolism through K63-linked ubiquitination of ECH1, highlights the importance of understanding the unique metabolic characteristics of GBM.

As a cytoplasmic adaptor of the TRAF family, TRAF3 functions as an important mediator of innate immune receptor signaling through its scaffolding function and E3 ubiquitin ligase activity in different immune cells, including B cells, T cells, and macrophages (15). Several studies have investigated the tumor-suppressive functions of TRAF3, including in B cell lymphoma and head and neck cancers (49, 50). An association study has also indicated that high promoter methylation of *TRAF3* correlates with the progression of cervical intraepithelial neoplasia (20). Moreover, TRAF3 deficiency has been reported to regulate metabolism reprogramming in B cells (51). TRAF3 is a multifaceted regulator with a range of mechanisms of action. Prior research has demonstrated that TRAF3 suppresses the alternative NF-κB pathways in glioma (52). Although NF-κB pathway inactivation has been demonstrated to significantly downregulate several enzymes involved in FAO, including Acyl-CoA dehydrogenase family member 9 (ACAD9), Acyl-CoA synthetase long-chain family member 4 (ACSL4), and FA desaturase 2 (FADS2) (53), our present study found that rescuing the alternative NF-κB pathway did not restore the decreased OCR caused by TRAF3 overexpression. This result suggests that TRAF3 regulates glioma metabolism through mechanisms that extend the NF-κB pathway. Furthermore, our findings indicate that, while overexpression of WT ECH1 did not significantly reverse the effect of TRAF3 overexpression on the accumulation of PUFAs, lipid metabolism, or GBM growth, transfection of the K214R-mutant ECH1 markedly reversed these effects. These findings provide compelling evidence that loss of TRAF3 critically regulates metabolic plasticity in glioma through the regulation of ECH1 ubiquitination.

Unlike SFAs that directly enter mitochondrial for β-oxidation, UFAs require specific enzymes to facilitate their complete oxidation. ECH1 is an essential enzyme that catalyzes isomerization of the 3,5-dienoyl-CoA substrate to 2,4-dienoyl-CoA, which is regarded as a crucial step in the reductase-dependent pathway because the 3,5-dienoyl-CoA substrate is a “dead-end metabolite” that is exclusively metabolized by ECH1 (25, 33). Consistent with the potential function of ECH1 in lipid metabolism, our study revealed that depletion of ECH1 led to impaired FAO, preferentially through accumulation of PUFAs in CL and TGs, resulting in oxidative mitochondrial damage and cell-growth arrest. These results highlight the importance of ECH1-mediated isomerization and metabolism of PUFAs in lipid peroxidation and GBM tumorigenesis. Notably, mRNA levels of *ECH1* were not significantly changed between low-grade glioma and GBM tumors (Supplemental Figure 5A). Thus, TRAF3-mediated K63 ubiquitination and mitochondrial translocation of ECH1 may play a vital role in regulating ECH1 activity and metabolic plasticity in GBM. Similarly, acetylation of enoyl-CoA hydratase, short chain 1 (ECHS1), another member of the enoyl-CoA hydratase family responsible for the second step of hydration of FAO, has been shown to impede its mitochondrial translocation and activity (54).

The responsiveness of tumors to immunotherapies is not only related to the cytotoxic activity of immune cells within the tumor microenvironment (TME), but also to the susceptibility of tumor cells to immune cell killing. Ferroptosis is a distinct form of programmed cell death characterized by iron accumulation and phospholipid peroxidation and has been proposed as a major type of cell death induced by immune cell–mediated cell killing, including by CD8^+^ T cells and neutrophils (39, 55). The present study demonstrated that TRAF3 overexpression in GBM cells did not directly induce ferroptosis or the cytotoxicity of CD8^+^ T cells. However, TRAF3-induced accumulation of unmetabolized PUFAs and lipid peroxidation led to increased vulnerability of GBM cells to ferroptosis induced by CD8^+^ T cells and sensitized GBM to anti–PD-L1 immunotherapy. Therefore, TRAF3/ECH1 axis–regulated PUFA metabolism controlled the energy supply on the one hand, and modulated the vulnerability of tumor cell to T cell–mediated cell killing on the other hand. Combination approaches that simultaneously target cancer metabolism and unleash the power of immunotherapy hold promise for improving patient outcomes in cancer treatment.

While the present study yielded promising findings, several limitations warrant further investigation in the future. First, although our study revealed that the TRAF3/ECH1 axis induced lipid peroxidation and subsequently increased the susceptibility of GBM cells to ferroptosis inducers, including anti–PD-L1 immunotherapy, direct mechanistic evidence linking lipid peroxidation with the efficacy of immunotherapy is required. Second, we did not investigate the direct effect of lipid accumulation on immune elimination of GBM cells in the context of TRAF3 overexpression. Accordingly, further research is necessary to examine the effect of lipid metabolism products on immune cells. Finally, the clinical application of manipulating the TRAF3/ECH1 axis is confronted with several challenges, including the efficient delivery of genes, the potential off-target effects, and the assurance of sustained expression levels. Recent advances in nanotechnology and material science have demonstrated the potential for developing efficient delivery systems for RNA and proteins targeting specific cells (56, 57). The insights gained from our study provide a foundation for future therapeutic strategies that will leverage advanced delivery systems and combination treatments.

In conclusion, our study demonstrated that loss of TRAF3 in GBM induced mitochondrial translocation of ECH1 and oxidation of PUFAs, thereby inhibiting lipid peroxidation, and promoted tumor growth. Our findings highlight a critical role of the TRAF3/ECH1 axis in the oxidation of PUFAs, and targeting the axis represents a promising strategy to repress GBM growth and enhance the vulnerability of GBM to immunotherapies.

## Methods

### Sex as a biological variable.

Clinical samples from patients of both sexes were included in this study. Nude mice and C57BL/6J mice of both sexes were used in this study.

### Statistical analysis.

Statistical analysis was performed using GraphPad Prism (GraphPad Software) or R software (R Foundation for Statistical Computing). Data are expressed as the mean ± SD. For representative data, the results were repeated in at least 3 independent experiments. For quantitative data, the statistical test used is indicated in the Figure legends. Statistical differences between 2 groups were analyzed by unpaired or paired, 2-tailed Student’s *t* test. One-way ANOVA followed by Tukey’s post hoc test was used to compare differences between multiple groups. Statistical significance of survival between groups was analyzed by log-rank test. A *P* value of less than 0.05 was considered as statistically significant.

### Study approval.

The care and use of animals for all animal experiments were approved by the IACUC of Southern Medical University. Anonymous archived human glioma specimens were obtained from the Department of Neurosurgery of Nanfang Hospital of Southern Medical University (Guangzhou, China) under a protocol approved by the IRB of that institution. Written informed consent was obtained from all participants.

### Data availability.

The raw RNA-Seq data for this study have been deposited in the Gene Expression Omnibus (GEO) database (GEO GSE285922). Values for all data points in graphs are reported in the Supporting Data Values file. The data generated in this study are available upon request from the corresponding author.

## Author contributions

AZ and YZ conceived the study. YZ, LZ, and KZ designed and performed most of the experiments. Ziling Zhan, PC, CX, SD, YX, SL, LW, CL, and XC assisted with some of the in vitro experiments. Zhengming Zhan and HZ assisted with the bioinformatics analysis. DG, EB, JL, FD, XB, and YS provided tissue samples, reagents, and conceptual advice. AZ and YZ wrote and revised the manuscript. AZ supervised the study. All authors discussed the results and commented on the manuscript.

## Supplementary Material

Supplemental data

Unedited blot and gel images

Supplemental table 1

Supplemental table 2

Supplemental table 3

Supplemental table 4

Supplemental table 5

Supplemental table 6

Supporting data values

## Version 1 02/11/2025

In-Press Preview

## Version 2 04/01/2025

Electronic publication
