# Supplementary material for: TRAF3 loss protects glioblastoma cells from lipid peroxidation and immune elimination via dysregulated lipid metabolism
Source: J Clin Invest. 2025 Feb 11;135(7):e178550. doi: 10.1172/JCI178550 (PMC11957706; doi:10.1172/JCI178550)
Supplement: Supplemental data [file jci-135-178550-s009.pdf]

Supplemental Information

TRAF3 Loss Protects Glioblastoma Cells from Lipid Peroxidation and Immune Elimination via Dysregulated Lipid Metabolism

Yu Zeng, Liqian Zhao, Kunlin Zeng, Ziling Zhan, Zhengming Zhan, Shangbiao Li, Hongchao Zhan, Peng Chai, Cheng Xie, Shengfeng Ding, Yuxin Xie, Li Wang, Cuiying Li, Xiaoxia Chen, Daogang Guan, Enguang Bi, Jianyou Liao, Fan Deng, Xiaochun Bai, Ye Song, Aidong Zhou

Supplemental Methods

Cell culture and treatment

Human GBM patient-derived cell line GBM0919, GBM0108 and GBM0709 were established from GBM tissues (WHO grade IV glioma) obtained during surgery in Nanfang Hospital, as we previously described (57). Briefly, tumor specimens were gently separated, minced and digested with collagenase (Sigma) to single cell suspension and then cultured in Dulbecco's modified Eagle medium (DMEM, Gibco) supplemented with 10% fetal bovine serum (FBS, Gibco) in a T75 cell culture flask (Corning). Only early-passage GBM cells were used for the study. The murine GBM cell lines CT-2A is a generous gift from Nanjing Medical University, and GL261, U87MG, LN229, T98G and SW1783 cells were kept in the lab. All cells were maintained under standard humidification conditions (37°C, 5% CO_2) with DMEM (Gibco) supplemented by 10% fetal bovine serum (Gibco) and 1% penicillin/streptomycin (Gibco).

For cell treatment, the standard concentrations of reagent used in this study, unless otherwise specified, are as follows: 5-azacytidine (5-Aza, Selleck), 5 μM; MG132 (Sigma), 20 μM; cycloheximide (CHX, Selleck), 50 μg/ml; glutathione ethyl ester (GSH-EE, Sigma), 3 mM; erastin (Sigma), 5 μM; Ferrostatin-1 (MCE), 10 μM; etomoxir (ETO, Sigma), 100 μM (a common concentration used in the literature to study CPT1a inhibition in glioma); linoleic acid-BSA (LA-BSA, Sigma), 100 μM.

Glioma Tissues

Anonymous archived human glioma specimens were obtained from Department of Neurosurgery of Nanfang Hospital of Southern Medical University (Guangzhou, China) under a protocol approved by the institutional review board. The paired NTB tissues were obtained similar to previous reports (58, 59). Specifically, normal-appearing brain tissue adjacent to the tumor tissue sample was carefully separated under microscopy and subsequently divided into fresh frozen tissues and formalin-embedded tissues. Hematoxylin-eosin (HE) staining was then performed and examined histopathologically by two neuropathologists independently to confirm the non-tumoral nature of the NTB tissues. All tissue samples were collected in compliance with the institution's informed consent policy. The patient information of NFYY glioma cohort was shown in Supplemental Table 6.

BSPCR and MSPCR analysis

Bisulfite sequencing polymerase chain reaction (BSPCR) and methylation-specific polymerase chain reaction (MSPCR) were performed to validate DNA methylation levels of *TRAF3* promoter region in GBM tissues and paired adjacent non-tumor tissues. The BSP-specific and MSP-specific primers were designed with MethPrimer. Bisulfide conversion was conducted using EZ DNA methylation kit (D5020, ZYMO Research) according to the manufacturer's protocol. Briefly, Genomic DNA from samples was extracted and then subjected to bisulfite modification, which induced the conversion of unmethylated cytosine and guanine (CG) sites to uracil-guanine (UG) pairs with no effect on methylated sites. PCR products were then cloned into T-vector and ten

randomly selected clones from each sample were sequenced for BSPPCR (Sangon Biotech, China) or were subject to electrophoresis for MSPCR analysis. All primers used for PCR are listed in Supplemental Table 7.

Plasmid construction

Full length CDS of human *TRAF3* and *ECH1* gene were amplified by RT-PCR from the cDNA library of SW1783 cells, and then were cloned into 3×Flag or pcDNA3-Myc vectors, respectively. Site-directed mutagenesis in ECH1 was introduced using the QuikChange site-directed mutagenesis kit (Agilent Technologies). For lentiviral expression, the CDSs of *TRAF3* and *ECH1* were further sub-cloned into pLVX-Neo or pLVX-Puro vector. The lentiviral shRNA expression plasmids were constructed by annealing the sense and antisense oligonucleotides of the target sequences, and the annealed oligos were then cloned into pLKO.1 vector. A plasmid expressing shRNA that targets no known gene were used as control. All plasmids were verified by DNA sequencing. Oligonucleotides for the construction of plasmids are shown in Supplemental Table 7.

Lentiviral stable cell lines

The lentiviral expression plasmids, packaging plasmid psPAX2, and envelope plasmid pMD2.G (4:3:1) were co-transfected into HEK293T cells using Lipofectamine 2000 reagent (Invitrogen, USA). Lentiviral particles in the cell culture medium were

collected 48 h after transfection, and then added onto GBM cells in six-well plates. The infected GBM cells were selected by puromycin (2 μg/ml) or neomycin (200 μg/ml) for 1 week.

Immunoprecipitation and mass spectrometry

Flag-TRAF3 plasmid or the control vector was transfected into U87MG cells, and cell lysates were immunoprecipitated with an antibody against Flag-Tag (Sigma-Aldrich). The isolated protein complexes were separated by sodium dodecyl sulfate polyacrylamide gel electrophoresis (SDS-PAGE), followed by sliver staining (ThermoFisher Scientific), according to the manufacturer's protocol. Protein bands of interest were excised and subjected to mass spectrometry analysis (Wininnovate Bio., Shenzhen). MS/MS spectra were searched against the UniProt Homo Sapiens Reference Proteome dataset using Mascot v2.5.1 (Matrix Science).

RNA extraction and transcriptome sequencing

Total RNA of GBM0709 cells overexpressing Flag-TRAF3 was extracted using Trizol reagent (Invitrogen) and quantified at an optical density of 260 nm (Nanodrop Technology). Total RNA Library were prepared using Illumina TruSeq RNA Sample Preparation kit, according to manufacturer's instruction. The paired-end reads were generated using the Illumina NovaSeq6000 platform (Berry Genomics, Beijing) and

then mapped to human genome (hg38) and normalized with *edgeR* package using TMM in R.

Quantitative RT-PCR

Extracted total RNA was reverse transcribed using PrimeScript RT reagent Kit (Takara) according to the manufacturer's instructions. The reverse-transcribed cDNA products were then used for qRT-PCR analysis with SYBR Premix Ex Taq (Takara) according to the manufacturer's instructions. GAPDH was used as an internal control. Primers were synthesized by Sangon Biotech (Shanghai), and the sequences are listed in Supplemental Table 7.

Isolation of mitochondrial proteins and immunoblotting

Mitochondrial and cytosolic fractions were isolated using the Mitochondria Isolation Kit for Cultured Cells (Pierce, 89874) according to the manufacturer's protocols. The isolated mitochondrial and cytoplasmic proteins were separated by SDS-PAGE and the analyzed by western blotting.

Cell viability assays

For cell viability analysis, the patient-derived GBM0709, GBM0108 or U87MG cells (1000 cells/well) overexpressing *TRAF3* or *ECH1* shRNAs were seeded in 96-well plates and grew in a 5% CO_2 incubator at 37°C for 7 days. The viability of cells was assessed by CCK8 assays (Vazyme Biotech, Nanjing, China), according to the

manufacturer's instructions. For colony formation assays, cells (1000 cells/well) were seeded in 6-well plates and grew in a 5% CO_2 incubator at 37°C for 10 days. Cells were stained with crystal violet, and the number of colonies in each well were counted.

Ubiquitination assays

For cellular ubiquitination, cells transfected with the indicated plasmids were treated with the proteasome inhibitor MG132 (20 μM) for 6 h, and then were lysed using RIPA lysis buffer (50 mM Tris-base pH 6.8, 150 mM NaCl, 1% NP-40, 0.5% deoxycholic acid, 0.1% SDS, 10 mM NaF, 10 mM DTT, 0.2 mM Na_3VO4, 1% cocktail protease inhibitors, and 1 mM PMSF). Cell lysates were incubated with the indicated antibodies and then washed for 3 times by RIPA buffer. To exclude non-specific ubiquitin-modified species from the ECH1 complex, the immunoprecipitated protein mixture was washed for 3 times using a ubiquitination wash buffer [50 mM Tris base pH 6.8, 150 mM NaCl, 1% NP-40, 0.5% deoxycholic acid, 1 M urea, 1 mM N-ethylmaleimide (NEM), and protease inhibitors].

For *in vitro* ubiquitination assays, purified wildtype ECH1 or ECH1-K214R (5 μM) was incubated with 2 μM TRAF3, 100 μM His-Ub or His-Ub-K63R (R&D Systems), 100 nM UBE1 (R&D Systems), 1 μM UBE2C (Novus), 10 mM Mg^{2+}-ATP in 50 μL reaction buffer (50 mM Tris PH 7.5, 5 mM $MgCl_2$ and 2 mM DTT) at 37°C for 1 h. The ubiquitinated proteins were purified by Ni-NTA beads (MedChemExpress), and the eluted proteins were analyzed by immunoblotting.

In vitro analysis of CD8⁺ T cell-mediated cytotoxicity

Human peripheral blood mononuclear cells (PBMCs) were isolated from healthy donor blood (LDEBIO, Guangzhou, China) by density gradient centrifugation. $CD8^+$ T cells were then purified from PBMCs using CD8 Dynabeads (Cat. 11147D, Invitrogen) following the manufacturer's instructions. Isolated $CD8^+$ cells were activated for 48 h with αCD3/αCD28 beads (Cat. 11132D, Gibco) and then cultured in the presence of human IL-2 (500 U/mL, R&D) for one week. For *in vitro* cytotoxicity assay, GBM cells were first plated on 96-well plates. After 24 h, activated $CD8^+$ T cells were added to the plates and co-cultured with the adherent GBM cells at increasing effector to target (E:T) ratios from 1:1 to 1:10 for 48 h. Cell viability was then determined using CCK8 assays and Calcein-AM/PI staining. The GZMB positive $CD8^+$ T cells was further determined by flow cytometry.

Metabolism analysis

Oxygen consumption rate (OCR) and extracellular acidification rate (ECAR) of tumor cells were measured by Seahorse XF96 Extracellular Flux Analyzer (Agilent) as described previously (60). Primary human GBM cells (20,000 cells/well) were plated on Seahorse culture plates (Agilent) in minimal Dulbecco's modified Eagle's medium (DMEM) supplemented with 25 mM glucose, 1 mM pyruvate and 2 mM glutamine. For mitochondrial stress tests, cells were treated with 2 μM oligomycin, 0.5 μM carbonyl cyanide 4-(trifluoromethoxy) phenylhydrazone (FCCP), 1 μM rotenone and 1

μM antimycin A during analysis, and OCRs and ECARs were then measured. Data were analyzed using Wave software (Agilent).

Measurement of intracellular ATP production

Intracellular ATP production was measured using the ATP assay kit (Beyotime, S0027) according to manufacturer's protocol. Cells were lysed in 200 μL lysis buffer and then centrifuged at 12,000 × g for 5 min at 4°C to collect the supernatant. An aliquot of 100 μL ATP detection working solution was added to a 96-well culture plate and was incubated for 5 min at room temperature. Then, the supernatant was added into the working solution, and the luminescence was measured immediately. The readout was normalized by the protein amounts of each sample.

Intracellular ROS, mitochondrial ROS, glutathione and malondialdehyde (MDA) measurement

Total intracellular and mitochondrial ROS levels were evaluated by flow cytometry using a FC500 flow cytometer (Beckman Coulter, USA) upon staining with CellROX Green or MitoSOX probe (Invitrogen, Shanghai, China) according to the manufacturer's instructions. The levels of reduced (GSH) and oxidized glutathione (GSSG) were measured with the GSH/GSSG Glo Assay (Promega, Beijing, China) according to the manufacturer's instructions.

Mitochondrial membrane potential

Mitochondrial membrane potential of GBM cells was evaluated by measuring JC-1 fluorescence with a mitochondrial membrane potential assay kit (M34152, Invitrogen). JC-1 fluorescence was observed by excitation at 488 nm and examination of the emission at 529 nm (JC-1 monomers) and 590 nm (JC-1 aggregates), respectively.

Transmission electron microscopy (TEM)

Samples were fixed with a fixation solution (3% glutaraldehyde, 2% paraformaldehyde in 0.1 mol/L cacodylate buffer, pH 7.3) for 1 h. After fixation, the samples were washed and treated with 0.1% Millipore-filtered cacodylate buffered tannic acid, postfixed with 1% buffered osmium tetroxide for 30 min, and stained with 1% Millipore-filtered uranyl acetate. The samples were dehydrated in increasing concentrations of ethanol, then infiltrated and embedded in LX-112 medium. The samples were polymerized in a 60°C oven for 2 days. Ultrathin sections were cut using a Leica Ultracut microtome, stained with uranyl acetate and lead citrate in a Leica EM Stainer, and examined in a JEM 1010 transmission electron microscope (JEOL) at an accelerating voltage of 80 kV. Digital images were obtained using an AMT Imaging System (Advanced Microscopy Techniques Corp.).

SA-β-gal staining

β-gal staining was performed using the senescence detection kit (QIA117-1 kit, Millipore), according to the manufacturer's instructions. Briefly, cultured GBM cells

or cryosections of mouse GBM tissues were incubated with staining solution for 16 h at 37°C, and then counterstained with eosin and mounted using 70% glycerin. Images were taken under a bright-field microscope.

Immunohistochemical (IHC) and Immunofluorescence (IF)

For immunohistochemical (IHC) staining, tissue slides were deparaffinized, rehydrated through an alcohol series, and then stained with primary antibodies against TRAF3, ECH1, Ki-67, 8-oxoG, 4-HNE, cleaved CASP3, or GZMB. Staining was scored on a scale from 0 to 12 according to the percentage of cells with positive staining and to the staining intensity, as we described previously (61). The stained tissue sections were reviewed and scored by 2 pathologists blinded to the clinical parameters.

For immunofluorescence (IF), cells in 6-well plates were treated with 4% formaldehyde for 15 min and then treated with 0.5% Triton X-100 for 15 min. The slides were incubated with antibodies against TRAF3, ECH1, TOMM20, HA-Tag, or Myc-Tag, and then incubated with a fluorescent-conjugated second antibody (Alexa Fluor, Life Technologies). For lipid peroxidation, GBM cells were treated with the lipid peroxidation probe C11 BODIPY 581/591 (1:1000; D3861, Thermo Fisher) for 15 min in the dark. The oxidized proportion was quantified as (average green intensity) / (average red intensity + average green intensity). The samples were mounted, and nuclei were visualized with DAPI-containing mounting media. Images were obtained by confocal microscopy (LSM800, Zeiss). Antibodies used in this study were shown in Supplemental Table 8.

Animals and in vivo intracranial GBM model

All procedures for rodent experiments and the determination of end points of animals were performed in accordance with the guidelines and protocols provided by the Southern Medical University Institutional Animal Care and Use Committee. Animals were randomized to different groups, and the investigators were blinded to allocation during experiments and outcome assessment. Human GBM cells (5×10^5 cells/mouse) or mouse CT-2A or GL261 GBM cells (1×10^5 cells/mouse) were intracranially injected into 4 to 6-week-old BALB/c nude (nu/nu) or C57BL/6 mice (5 mice for each group). At the appearance of serious neurological symptoms and significant weight loss, mice were anesthetized and sacrificed, and each mouse's brain was harvested, fixed in 4% formaldehyde, and embedded in paraffin. Tumor formation was determined by histologic analysis of tissue sections stained with hematoxylin and eosin (H&E). Tumor volume was calculated according to the formula $V = \pi/6 \times \text{length} \times \text{width}^2$.

For *in vivo* therapeutic experiments, 6 d after intracranial implantation of GBM cells into BALB/c nude, mice were intraperitoneally injected with erastin (15 mg/kg/d) every other day for four times. For anti-PD-L1 immunotherapy, after implantation of CT-2A or GL261 cells into C57BL/6 mice, mice were intraperitoneally injected with an anti-PD-L1 mAb (200 μg/mouse; Clone: 10F.9G2, BE0101, BioXcell) for two times. A CD8α depleted mouse model was established as previously described (62). Briefly, 150 μg of anti-mouse CD8α mAb (Clone: 2.43, BE0061, BioXcell) was diluted in 200 μL of PBS and injected intraperitoneally into each mouse. The initial intraperitoneal

injection was performed one day before GL261 cells injection, with additional injections 7 and 14 days thereafter, respectively. For survival analysis, the survival days of mice in each group were recorded when the mice died.

Flow cytometry

Single cell suspension of GL261 tumors were prepared as we previous reported (61). Cells were then stained with PE/Cyanine7-CD45, PerCP/Cyanine5.5-CD3, and APC-CD8 (BioLegend) for 30 min. Following fixation and permeabilization by Fixation Buffer and Intracellular Staining Perm Wash Buffer (BioLegend), intracellular GZMB was stained using FITC-GZMB antibody. The stained cells were then analyzed using a DxP Athena flow cytometry system (Cytek Biosciences, USA), with data processing performed by CytExpert and FlowJo software.

Bioinformatic analysis

RNA expression and corresponding clinicopathological data of LGG and GBM patients were obtained from the TCGA (http://xena.ucsc.edu/), CGGA (https://cgga.org.cn), and CPTAC (https://cptac-data-portal.georgetown.edu/study-summary/S048) database. Standardized pan-cancer data set (PANCAN, N=10535, G=60499) was obtained from the UCSC (https://xenabrowser.net/) database. We further extracted expression data of TRAF family members in each sample, with a $\log_2(x+0.001)$ transformation on each expression value. In addition, TCGA prognostic data was obtained from previous study (63). Cox proportional hazards regression model was established based on overall

survival for each cancer species with *survival* package (version 3.2-7) in R. Differentially-expressed genes (DEGs) analysis was conducted with *limma* package, and GSEA was performed with *clusterProfiler* package in R.

For single-cell RNA-seq analysis, normalized glioma single-cell RNA-seq data was downloaded (SCP1985). Based on the original cell annotations, the most abundant cell subpopulations including tumor cells, myeloid cells, lymphoid cells, and oligodendrocytes were extracted for subsequent analysis. Cells with TRAF3 expression greater than 0 were defined as TRAF3-positive. The positive score was calculated based on the positive TRAF3 expression rate of the 20 closest neighboring cells in the principal component space for each individual cell. A Chi-squared test was conducted on the proportion of positive expression in each cell subpopulation.

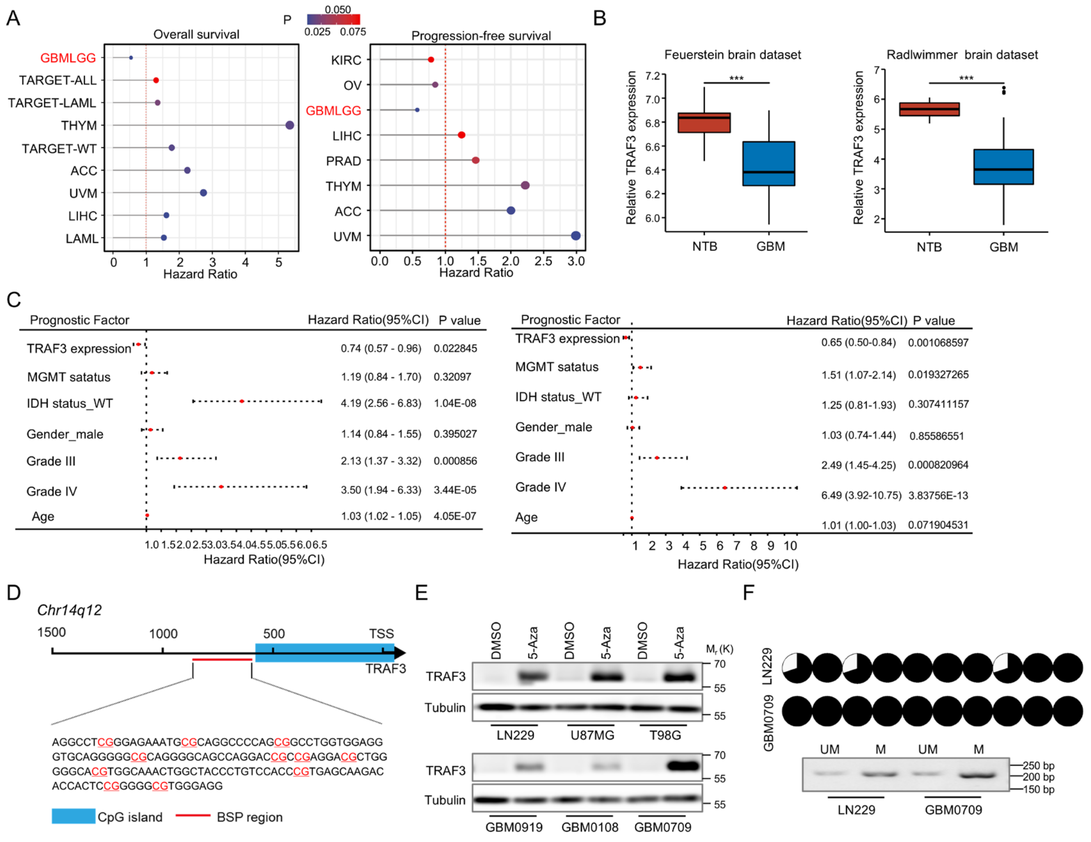

Supplemental Figure 1. Loss of *TRAF3* expression by hypermethylation correlates with poor prognosis in glioma. (A) Multivariate cox regression analysis shows the correlation of *TRAF3* levels with patient survival in different cancer types. **(B)** The levels of *TRAF3* in non-tumor brain (NTB) tissues and different grades of gliomas from the Feuerstein brain dataset and Radlwimmer brain dataset, respectively. ***P<0.001. **(C)** Multivariate cox regression analysis shows that *TRAF3* expression is an independent predictor of favorable prognosis in glioma in both TCGA (left panel) and CGGA (right panel) datasets, respectively. **(D)** The genome sequence of CpG island and Bisulfite sequencing polymerase chain reaction (BSP) region in *TRAF3* gene promoter. **(E)** GBM cells were treated with DMSO or 5-azacytidine (5-Aza), and TRAF3 protein were detected by immunoblotting. **(F)** BSP (upper panel) and methylation-specific PCR (MSP) analysis (lower panel) were used to analyze the methylation of *TRAF3* promoter in LN229 and GBM0709 cells. Statistical analysis was performed using unpaired t-test (B).

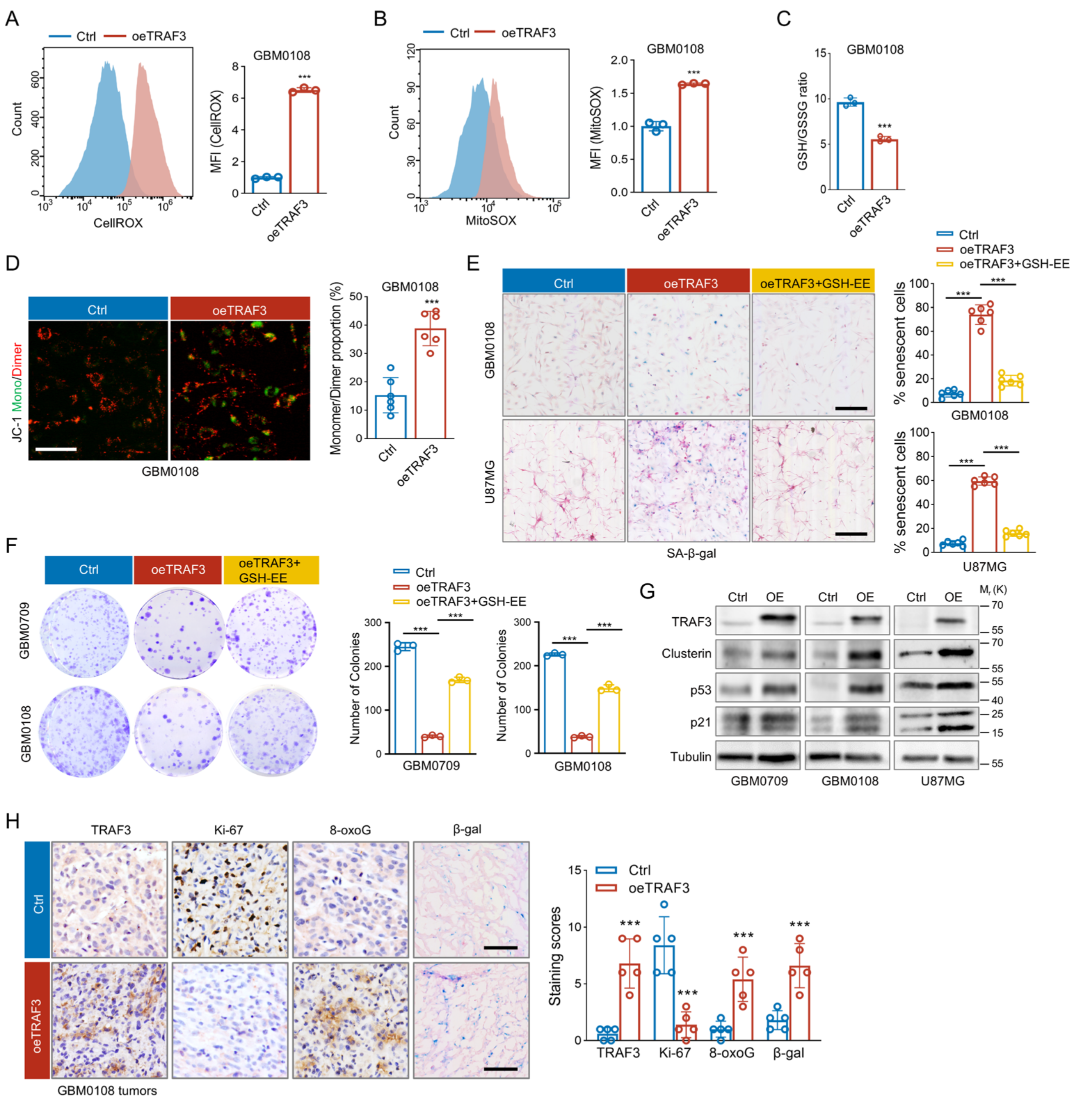

Supplemental Figure 2. TRAF3 overexpression promotes ROS-related mitochondrial dysfunction and inhibits GBM tumorigenesis. (A and B) Cellular (CellROX) and mitochondrial (MitoSOX) ROS levels of GBM0108 cells expressing TRAF3 were analyzed by flow cytometry. Representative flow cytometry plots were shown. The CellROX **(A)** and MitoSOX **(B)** levels were statistically analyzed (mean ± SD, n=3 independent experiments). ***P<0.001. **(C)** The reduced glutathione/oxidized glutathione (GSH/GSSG) ratios in GBM0108 cells expressing TRAF3 were measured (mean ± SD, n=3 independent experiments). ***P<0.001. **(D)** JC-1 staining of GBM0108 cells overexpressing *TRAF3*. Representative images were shown, Scale bar, 100 μm. The monomer/dimer ratios of JC-1 were statistically analyzed (mean ± SD, n=6 randomly selected microscope fields). ***P<0.001. **(E)** GBM0108 and U87MG cells expressing TRAF3 were treated with GSH-EE or not, and then stained with β-gal. Representative images were shown. Scale bars, 100 μm. The percentages of β-gal positive cells were counted (mean ± SD, n=6 randomly selected microscope fields). ***P<0.001. **(F)** GBM0709 and GBM0108 cells expressing TRAF3 were treated with GSH-EE or not, and cell survival was analyzed by colony formation assays (mean ±

SD, n=3 independent experiments). ***P<0.001. **(G)** GBM0709 and GBM0108 cells expressing TRAF3 were treated with GSH-EE or not, and cell lysates were analyzed by immunoblotting using the indicated antibodies. **(H)** Mouse GBM tissues derived from GBM0108 cells were stained with TRAF3, Ki-67, 8-oxoG, and β-gal, respectively. Scale bars, 100 μm. The staining scores in those two groups were compared with each other (mean ± SD, n=5 randomly selected microscope fields). ***P<0.001. Statistical analysis was performed using the unpaired t-test (A-D and H) or one-way ANOVA with Tukey post hoc test (E and F).

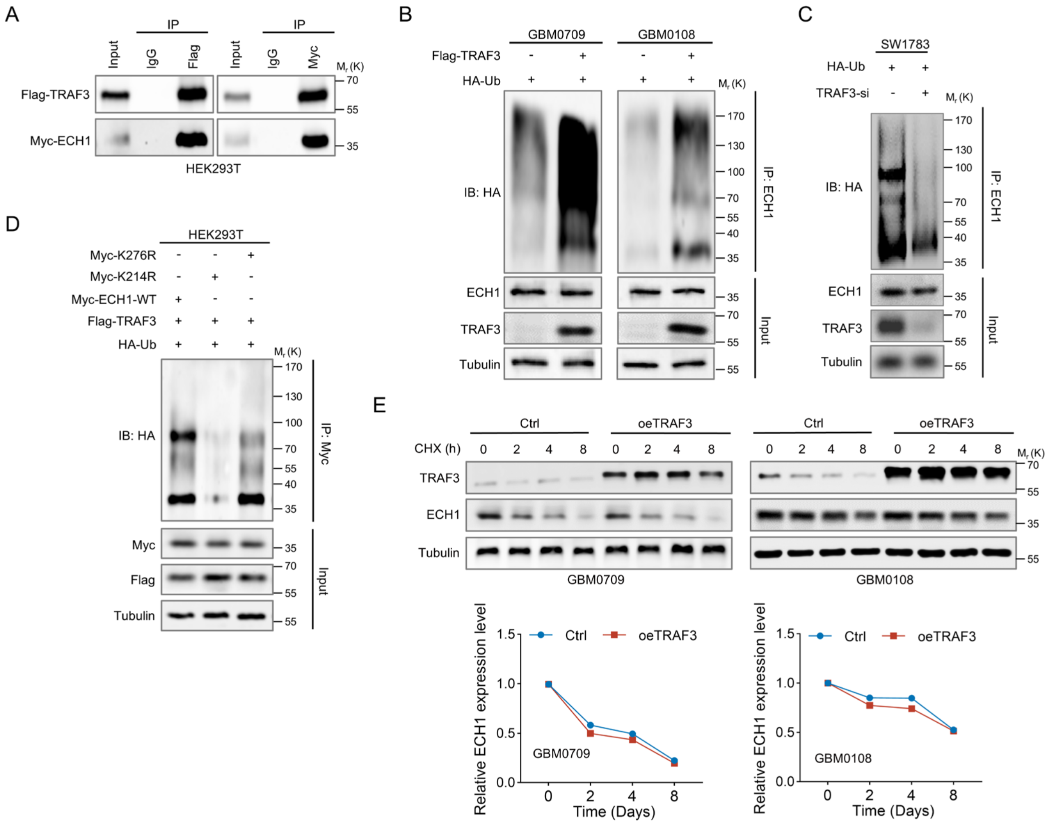

Supplemental Figure 3. TRAF3 interacts with ECH1 and promotes K63-linked ubiquitination of ECH1 at Lys214. (A) HEK293T cells were transfected with Flag-TRAF3 and Myc-ECH1 plasmids, and the cell lysates were immunoprecipitated using the indicated antibodies and then analyzed by immunoblotting. **(B)** GBM0709 and GBM0108 cells were transfected with Flag-TRAF3 and HA-Ub, and the cell lysates were immunoprecipitated with an anti-ECH1 antibody and then analyzed by immunoblotting. **(C)** SW1783 glioma cells were transfected with *TRAF3* siRNA and HA-Ub, and cell lysates were immunoprecipitated with an anti-ECH1 antibody and then analyzed by immunoblotting. **(D)** HEK293T cells were transfected with Flag-TRAF3, HA-Ub, and Myc-ECH1-WT/K276R/K214R, and cell lysates were immunoprecipitated with an anti-ECH1 antibody and then analyzed by immunoblotting. **(E)** GBM0709 and GBM0108 cells were transfected with Flag-TRAF3 and then treated with cycloheximide (CHX) for the indicated time intervals. Cell lysates were subjected to immunoblotting analysis using the indicated antibodies. The intensities of bands were quantified, and the results were expressed as ECH1 levels relative to control (mean ± SD, n=3 independent experiments, one-way ANOVA test).

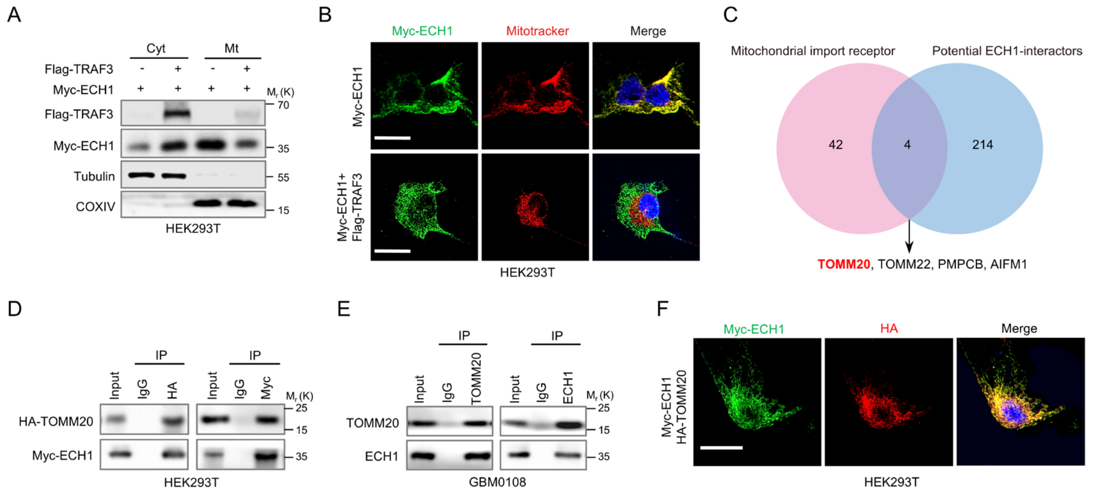

Supplemental Figure 4. TRAF3-mediated ubiquitination of ECH1 inhibits its mitochondrial translocation. (A) HEK293T cells were transfected with Myc-ECH1 and Flag-TRAF3, and the cytosolic and mitochondrial protein fractions were isolated and then were subjected into immunostaining using the indicated antibodies. **(B)** HEK293T cells were transfected with Myc-ECH1 and Flag-TRAF3, and cells were analyzed by immune staining. Scale bars, 20 µm. **(C)** Four proteins associated with mitochondrial transport were predicted to interact with ECH1 using the BioGRID protein interaction database. **(D)** HEK293T cells were transfected with Myc-ECH1 and HA-TOMM20, and the cell lysates were immunoprecipitated with an anti-HA or anti-Myc antibody and then analyzed by immunoblotting. **(E)** Co-immunoprecipitation assays demonstrated the reciprocal interaction between ECH1 and TOMM20 in GBM0108 cells. **(F)** IF staining demonstrated the co-localization between ECH1 and TOMM20 in HEK293T cells. Scale bar, 20 µm.

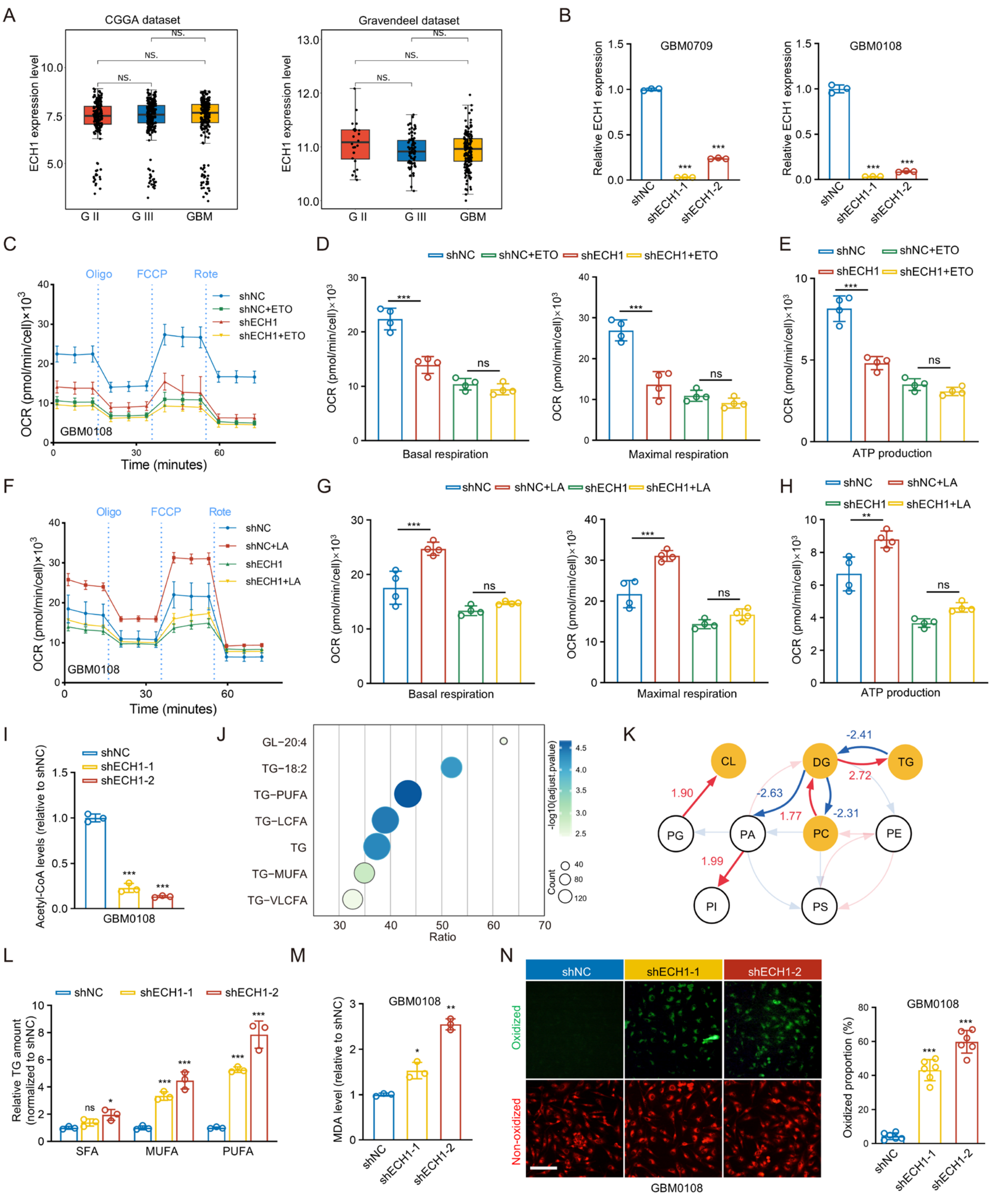

Supplemental Figure 5. ECH1 depletion induces accumulation of PUFAs and lipid peroxidation. (A) *ECH1* mRNA levels in different grades of gliomas were analyzed using the CGGA datasets and Gravendeel datasets. ns, not significant. **(B)** The mRNA levels of *ECH1* in GBM0709 and GBM0108 cells expressing *ECH1* shRNAs were examined by qRT-PCR (mean ± SD, n=3 independent experiments). ***P<0.001. **(C)** GBM0108 cells stably expressing *ECH1* shRNA were treated with etomoxir (ETO), and time series of oxygen consumption rate (OCR) was measured by Seahorse assays. **(D)** Quantification of basal respiration and maximum respiration in GBM0108 cells. **(E)** Quantification of mitochondrial ATP production in GBM0108 cells. **(F)** GBM0108 cells stably expressing *ECH1* shRNA were treated with BSA-conjugated linoleic acid (LA-BSA), and time series of oxygen consumption rate (OCR) was measured by

Seahorse assays. **(G)** Quantification of basal respiration and maximum respiration in GBM0108 cells. **(H)** Quantification of mitochondrial ATP production in GBM0108 cells. **(I)** Quantification of acetyl-CoA levels in GBM0108 cells. From **(C)** to **(I)**, data were expressed as mean ± SD of n=3 or 4 independent experiments. ns, not significant. **P<0.01, ***P<0.001. **(J)** Enrichment analysis of downregulated lipid species in GBM0709 cells expressing shECH1-2 compared to control shRNA. **(K)** Biosynthetic analysis of lipid species in GBM0709 cells expressing shECH1-2 compared to control shRNA. **(L)** Analysis of the content of SFAs, MUFAs, and PUFAs in TG in GBM0709 cells expressing *ECH1* shRNA compared to control shRNA (mean ± SD, n=3 independent experiments). ns, not significant. *P<0.05, ***P<0.001. **(M)** The levels of malondialdehyde (MDA) were detected in GBM0108 cells expressing *ECH1* shRNAs (mean ± SD, n=3 independent experiments). *P<0.05, **P<0.01. **(N)** BODIPY 581/591 staining in GBM0108 cells expressing control shRNA or *ECH1* shRNAs. Representative images were shown. Scale bars, 100 µm. The proportion of oxidized cells were calculated (mean ± SD, n=6 randomly selected microscope fields). ***P<0.001. Statistical analysis was performed using one-way ANOVA with Tukey post hoc test (A, B, D, E, G, H, I, L, M, and N).

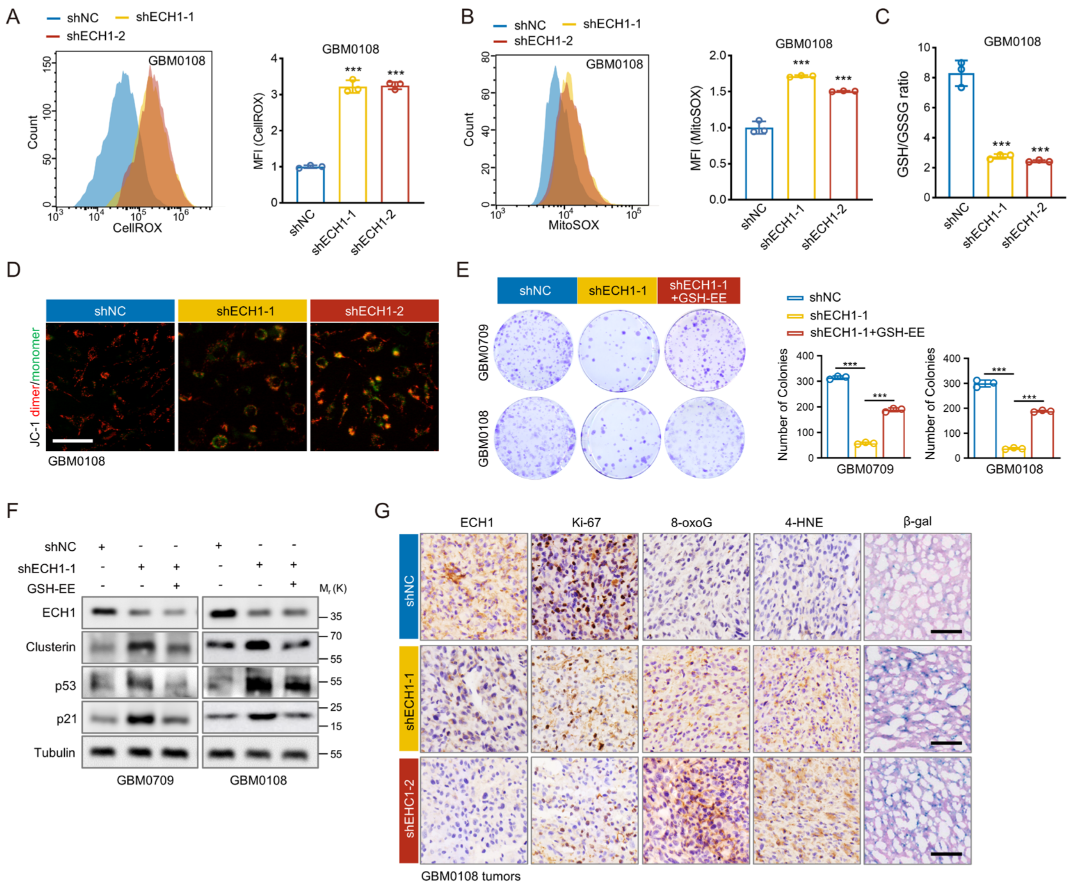

Supplemental Figure 6. ECH1 depletion triggers ROS-related mitochondrial damage and inhibits GBM tumorigenesis. (A and B) Flow cytometry analysis of CellROX **(A)** and MitoSOX staining **(B)** in GBM0108 cells stably expressing *ECH1* shRNAs. Representative flow cytometry plots were shown. The median fluorescence intensity (MFI) were quantified (mean ± SD, n=3 independent experiments). ***P<0.001. **(C)** GSH/GSSG ratios were evaluated in GBM0108 cells stably expressing *ECH1* shRNAs (mean ± SD, n=3 independent experiments). ***P<0.001. **(D)** JC-1 staining in GBM0108 cells expressing *ECH1* shRNAs. Representative images were shown. Scale bar, 100 μm. **(E)** GBM0709 and GBM0108 cells expressing *ECH1* shRNAs were treated with GSH-EE or note, and cell survival was analyzed by colony formation assays. Representative images were shown. The number of colonies were counted (mean ± SD, n=3 independent experiments). ***P<0.001. **(F)** GBM0709 and GBM0108 cells expressing *ECH1* shRNAs were treated GSH-EE or not, and the cell lysates were analyzed by immunoblotting using the indicated antibodies. **(G)** Mouse tumor tissues derived from GBM0108 cells were stained with ECH1, Ki-67, 8-oxoG, ,4-HNE, and β-gal, respectively. Scale bars, 200 μm. Statistical analysis was performed using one-way ANOVA with Tukey post hoc test (A, B, C, and E).

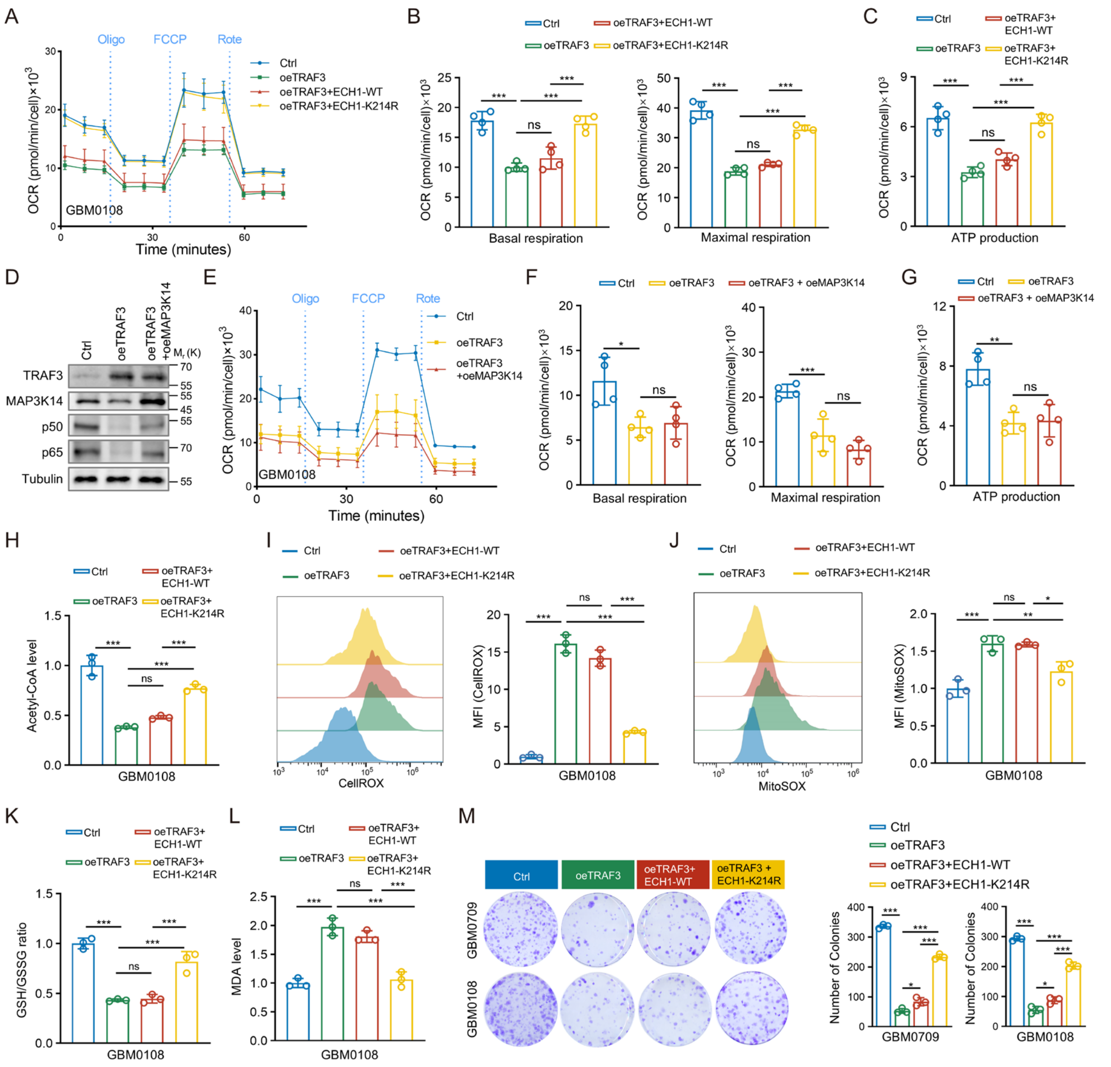

Supplemental Figure 7. TRAF3 overexpression impedes FAO and induces lipid peroxidation in GBM cells through ubiquitination of ECH1. (A) Time series of oxygen consumption rate (OCR) measurement in GBM0108 cells overexpressing TRAF3, TRAF3+ECH1-WT, or TRAF3+ECH1-K214R using the Seahorse Metabolic Analyzer. **(B)** Quantification of basal respiration and maximum respiration in GBM0108 cells. **(C)** Quantification of mitochondrial ATP production in GBM0108 cells. From **(A)** to **(C)**, data were expressed as mean ± SD of n=4 independent experiments. ns, not significant. ***P<0.001. **(D)** GBM0108 cells expressing TRAF3 were transfected with either a MAP3K14 overexpression plasmid or a control plasmid, and the cell lysates were analyzed by immunoblotting using the indicated antibodies. **(E)** Time series of OCR measurement in GBM0108 cells overexpressing TRAF3 and TRAF3+MAP3K14 using the Seahorse Metabolic Analyzer. **(F)** Quantification of basal respiration and maximum respiration in GBM0108 cells. **(G)** Quantification of mitochondrial ATP production in GBM0108 cells. **(H)** Quantification of acetyl-CoA levels in GBM0108 cells. From **(E)** to **(H)**, data were expressed as mean ± SD of n= 4 independent experiments. ns, not significant. *P<0.05, **P<0.01, ***P<0.001. **(I and J)** Flow cytometry analysis of CellROX **(I)** and MitoSOX **(J)** in GBM0108 cells

overexpressing TRAF3, TRAF3+ECH1-WT, or TRAF3+ECH1-K214R. Representative flow cytometry plots were shown. The median fluorescence intensities (MFI) were quantified (mean ± SD, n=3 independent experiments). ns, not significant. *P<0.05, **P<0.01, ***P<0.001. **(K)** GSH/GSSG ratios were evaluated in GBM0108 cells overexpressing TRAF3, TRAF3+ECH1-WT, or TRAF3+ECH1-K214R. **(L)** The levels of malondialdehyde (MDA) were detected in GBM0108 cells overexpressing TRAF3, TRAF3+ECH1-WT, or TRAF3+ECH1-K214R. In **(K)** and **(L)**, data were expressed as mean ± SD of n=3 independent experiments. ns, not significant. ***P<0.001. **(M)** Cell survival of GBM0709 and GBM0108 cells stably expressing TRAF3, TRAF3+ECH1-WT, or TRAF3+ECH1-K214R was analyzed by colony formation assays. The number of colonies were counted (mean ± SD, n=3 independent experiments). *P<0.05, ***P<0.001. Statistical analysis was performed using one-way ANOVA with Tukey post hoc test (B, C, F, G, H, I, J, K, L, and M).

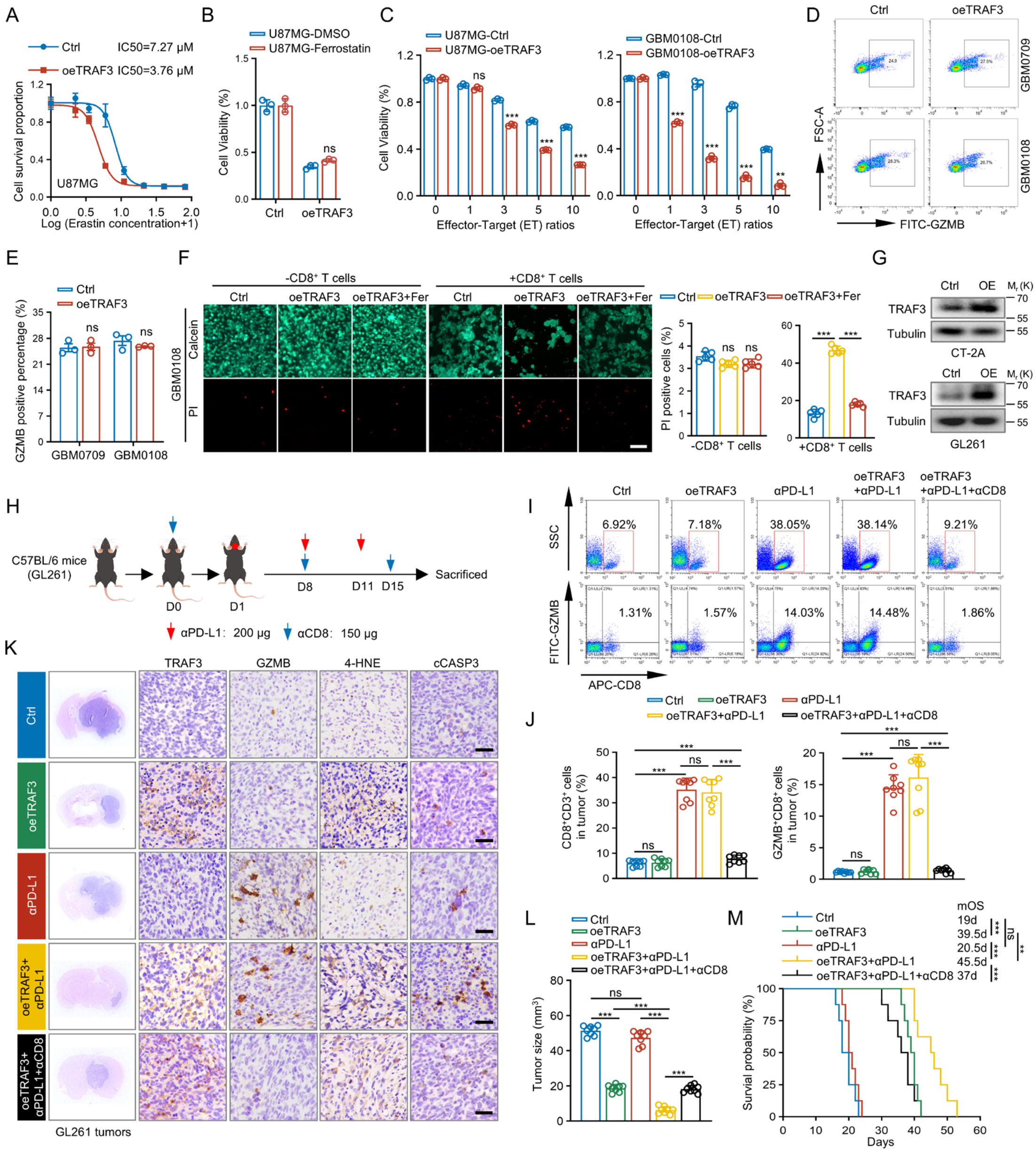

Supplemental Figure 8. Overexpression of TRAF3 sensitizes GBM to erastin-induced ferroptosis and anti-PD-L1 therapy. (A) U87MG cells expressing TRAF3 were treated with different concentration of erastin for 72 h. IC_{50} value was calculated (mean ± SD, n=4 independent experiments). **(B)** U87MG cells expressing TRAF3 were treated with Ferrostatin for 48 h, and cell viabilities were analyzed (mean ± SD, n=3 independent experiments). ns, not significant. **(C)** U87MG and GBM0108 cells stably expressing TRAF3 were co-cultured with activated $CD8^+$ T cells for 48 h with different effector-target (E-T) ratios (from 1:1 to 10:1). Cell viabilities after co-culture were evaluated (mean ± SD, n=3 independent experiments). ns, not significant. **P<0.01, ***P<0.001. **(D-E)** GBM0709 and GBM0108 cells expressing TRAF3 were co-cultured with activated $CD8^+$ T cells for 48 h (E-T ratio=3:1). After that, the T cells were subjected to flow cytometry analysis. Representative flow cytometry plots (D) and statistical quantitation of $GZMB^+$ cells (**E**) were shown (mean ± SD, n=3

independent experiments). ns, not significant. **(F)** GBM0108 cells expressing TRAF3 were treated with Ferrostatin or DMSO and then co-cultured with activated $CD8^+$ T cells for 48 h (E-T ratio=3:1). The surviving and dead cells were stained by Calcein-AM/PI. Representative images were shown. Scale bars, 100 μm. The percentage of PI positive cells were counted (mean ± SD, n=5 randomly selected microscope fields). ns, not significant. ***P<0.001. **(G)** Cell lysates of CT-2A and GL261 cells expressing TRAF3 were analyzed by immunoblotting. **(H)** Schematic representation of mouse treatment schedules. GL261 cells stably expressing *TRAF3* were intracranially injected into C57BL/6 mice. For the αCD8 depleted treatment, 150 μg of αCD8 was injected intraperitoneally into each mouse one day before GL261 cells injection, with additional injections 7 and 14 days thereafter. Mice were then intraperitoneally injected with αPD-L1 (200 μg/mouse/d) or control for two times. **(I-J)** Representative flow cytometry plots and quantification of $CD8^+$ and $GZMB^+CD8^+$ cells in mouse brain tissues (mean ± SD, n=8 mice for each group). ns, not significant. ***P<0.001. **(K)** H&E-stained sections show representative tumor xenografts. The mouse tumor tissues were stained with TRAF3, GZMB, 4-HNE, and cleaved-CASP3, respectively. Scale bars, 100 μm. **(L)** Tumor volumes were calculated (mean ± SD, n=8 mice for each group). ns, not significant. ***P<0.001. **(M)** The survival of the GL261 GBM-bearing mice was evaluated (n=8 mice for each group, Kaplan-Meier model). ns, not significant. **P<0.01, ***P<0.001. Statistical analysis was performed using one-way ANOVA with Tukey post hoc test (C, F, J, and L), unpaired t-test (B and E), or Log-rank test (M).

Supplemental Table 1-6 were uploaded as independent files.

Supplemental Table 1 RNA-seq analysis of GBM cells following TRAF3 overexpression.

Supplemental Table 2 GSEA analysis shows the enriched pathways following the overexpression of TRAF3.

Supplemental Table 3 Identification of TRAF3 interacting proteins by IP-MS/MS.

Supplemental Table 4 Lipidomic analysis of GBM cells following ECH1 depletion.

Supplemental Table 5 Lipidomic analysis of GBM cells with reconstituted expression of wild-type or K214R mutant ECH1 following TRAF3 overexpression.

Supplemental Table 6 Patient information of the NFYY glioma cohort.

Supplemental Table 7 Oligonucleotides used in the study.

Name	Sequence (5'-3')
TRAF3-Human-OE	F: 5'-AGGTCGACTCTAGAGGATCCCGCCACCATGGAG TCGAGTAAAAAGATG-3'
	R: 5'-TCCTTGTAGTCCATACCGGGATCGGGCAGATCC GAAGTATC-3'
ECH1-Human-sh1	F: 5'-CCGGGTTCCCAGACAAAGAGGTCATCTCGAGAT GACCTCTTTGTCTGGGAACTTTTTG-3'
	R: 5'-AATTCAAAAAGTTCCCAGACAAAGAGGTCATCT CGAGATGACCTCTTTGTCTGGGAAC-3'

ECH1-Human-sh2	F: 5’-CCGGGTAGAGTGCTTCAACAAGATTCTCGAGAATCTTGTTGAAGCACTCTACTTTTTG-3’
	R: 5’-AATTCAAAAAGTAGAGTGCTTCAACAAGATTCTCGAGAATCTTGTTGAAGCACTCTAC-3’
Primer for PCR	
Flag-TRAF3	F: 5’-CCCAAGCTTATGGAGTCGAGTAAAAAGATG-3’
	R: 5’-CGGGATCCTCAGGGATCGGGCAGATCCG-3’
Myc-ECH1	F: 5’-ACGGGCCCTCTAGACTCGAGCGCCACCATGGCGGCGGGGATAGTGGC-3’
	R: 5’-TTAAACTTAAGCTTGGTACCTCACAGATCCTCTTCTGAGATGAG-3’
HA-TOMM20	F: 5’-CCCAAGCTTATGGTGGGTCGGAACAGC-3’
	R: 5’-CGGGATCCTCATTCCACATCATCTTCAGC-3’
BSP-Inner pair	F: 5’-AGGTTTAGTTAGGTTGGGTG-3’
	R: 5’-AATAACACTTTCCTTTCTCCC-3’
BSP-Outer pair	F: 5’-GTTATTGGAGTAATATAAGAAGGTAGA-3’
	R: 5’-AAAAACAAAACTCCACATTA-3’
MSP-primer-M	F: 5’-GTAGTTAGGATCGTCGAGGAC-3’
	R: 5’-AAAACTAAAAATAACTCAAACCGTA-3’
MSP-primer-U	F: 5’-GTAGTTAGGATTGTTGAGGATGT-3’
	R: 5’-AAAACTAAAAATAACTCAAACCATA-3’

Primer for RT-qPCR	
Human-TRAF3	F: 5’-GCGTGTCAAGAGAGCATCGTT-3’
	R: 5’-GCAGATGTCCCAGCATTAACT-3’
Human-ECH1	F: 5’-TCATCACTCGATACCAGGAGAC-3’
	R: 5’-GGCACAGTACCGGATGTCA-3’
Human-MAPK11	F: 5’-AAGCACGAGAACGTCATCGG-3’
	R: 5’-TCACCAAGTACACTTCGCTGA-3’
Human-CBX2	F: 5’-GCCCAGCACTGGACAGAAC-3’
	R: 5’-CACTGTGACGGTGATGAGGTT-3’
Human-FOS	F: 5’-CACTCCAAGCGGAGACAGAC-3’
	R: 5’-AGGTCATCAGGGATCTTGCAG-3’
Human-MDM2	F: 5’-GAATCATCGGACTCAGGTACATC-3’
	R: 5’-TCTGTCTCACTAATTGCTCTCCT-3’
Human-TP53	F: 5’-ACTTGTCGCTCTTGAAGCTAC-3’
	R: 5’-GATGCGGAGAATCTTTGGAACA-3’
Human-TNR6C	F: 5’-CGTCCAAAGCCCTTCTAATCAG-3’
	R: 5’-TCTCCCATCTCCAATCATAGTGT-3’
Human-GAPDH	F: 5’-ACAACTTTGGTATCGTGGAAGG-3’
	R: 5’-GCCATCACGCCACAGTTTC-3’
siRNA	
TRAF3-siRNA-1	GUGAUGAUACAGGAGAAAU

TRAF3-siRNA-2	GUUGUGCAGAGCAGUUAAU
TOMM20-siRNA-1	CCUUACCUGACUAAUUGUU
TOMM20-siRNA-2	GCUGUUCAGAAGUUCUUCC

Supplemental Table 8 Information of antibodies used in the study.

Antibody	Company	Cat.no	Dilution
TRAF3	Proteintech	18099-1-AP	WB: 1:1000 IP: 1:500 IHC: 1:100
Tubulin	Ray Antibody	RM2003	WB: 1:5000
Ki-67	ZSGB-Bio	ZM-0166	IHC: 1:100
8-oxoG	Bioss	Bs-1278R	IHC: 1:100
Clusterin	Abcam	ab42673	WB: 1:1000
p53	Proteintech	10442-1-AP	WB: 1:1000
p21	SantaCruz	sc-397	WB: 1:1000
Flag	Proteintech	20543-1-AP	WB: 1:2000 IP 1:500
IgG	CST	2729	IP 1:500
ECH1	Proteintech	11385-1-AP	WB: 1:500 IP: 1:500 IHC/IF: 1:100
Myc	Proteintech	60003-2-Ig	WB: 1:5000 IP 1:500 IF: 1:200
HA	Proteintech	51064-2-AP	WB: 1:5000 IP 1:500
Ub	Proteintech	10201-2-AP	WB: 1:1000
GAPDH	Ray Antibody	RM2002	WB: 1:5000
COXIV	Proteintech	11242-1-AP	WB: 2:1000
TOMM20	Proteintech	11802-1-AP	WB: 1:1000 IP: 1:500 IF: 1:200

4-HNE	R&D Systems	MAB3249-SP	IHC: 1:500
GZMB	Proteintech	13588-1-AP	IHC: 1:100
cCASP3	Immunoway	YM3431	IHC: 1:100
PE/Cy7-CD45	Biolegend	103114	FC: 0.5 μg per million cells
PerCP/Cy5.5-CD3	Biolegend	100218	FC:1 μg per million cells
APC-CD8	Biolegend	100712	FC: 0.25 μg per million cells
FITC-GZMB	Biolegend	372206	FC: 0.25 μg per million cells

Supplemental References

1. Li S, Chen Y, Xie Y, Zhan H, Zeng Y, Zeng K, et al. FBXO7 Confers Mesenchymal Properties and Chemoresistance in Glioblastoma by Controlling Rbfox2-Mediated Alternative Splicing. *Adv Sci (Weinh).* 2023:e2303561.
2. Liu YY, Chen MB, Cheng L, Zhang ZQ, Yu ZQ, Jiang Q, et al. microRNA-200a downregulation in human glioma leads to Galphai1 over-expression, Akt activation, and cell proliferation. *Oncogene.* 2018;37(21):2890-902.
3. Nakamura M, Liu T, Husain S, Zhai P, Warren JS, Hsu CP, et al. Glycogen Synthase Kinase-3alpha Promotes Fatty Acid Uptake and Lipotoxic Cardiomyopathy. *Cell Metab.* 2019;29(5):1119-34 e12.
4. Zeng K, Zeng Y, Zhan H, Zhan Z, Wang L, Xie Y, et al. SEC61G assists EGFR-amplified glioblastoma to evade immune elimination. *Proc Natl Acad Sci U S A.* 2023;120(32):e2303400120.
5. Kim J, Kim Y, La J, Park WH, Kim HJ, Park SH, et al. Supplementation with a high-glucose drink stimulates anti-tumor immune responses to glioblastoma via gut microbiota modulation. *Cell Rep.* 2023;42(10):113220.
6. Liu J, Lichtenberg T, Hoadley KA, Poisson LM, Lazar AJ, Cherniack AD, et al. An Integrated TCGA Pan-Cancer Clinical Data Resource to Drive High-Quality Survival Outcome Analytics. *Cell.* 2018;173(2):400-16 e11.
