# Supplementary material for: TRAF3 loss protects glioblastoma cells from lipid peroxidation and immune elimination via dysregulated lipid metabolism
Source: J Clin Invest. 2025 Feb 11;135(7):e178550. doi: 10.1172/JCI178550 (PMC11957706; doi:10.1172/JCI178550)

Figure 1

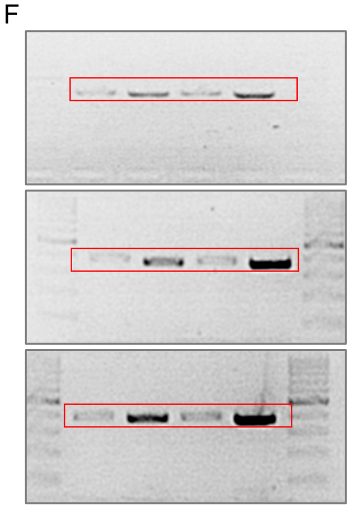

Figure 3

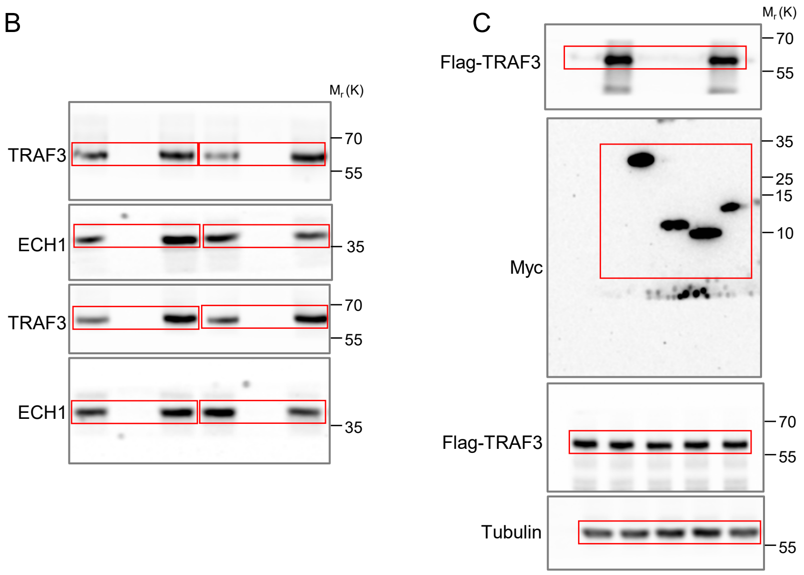

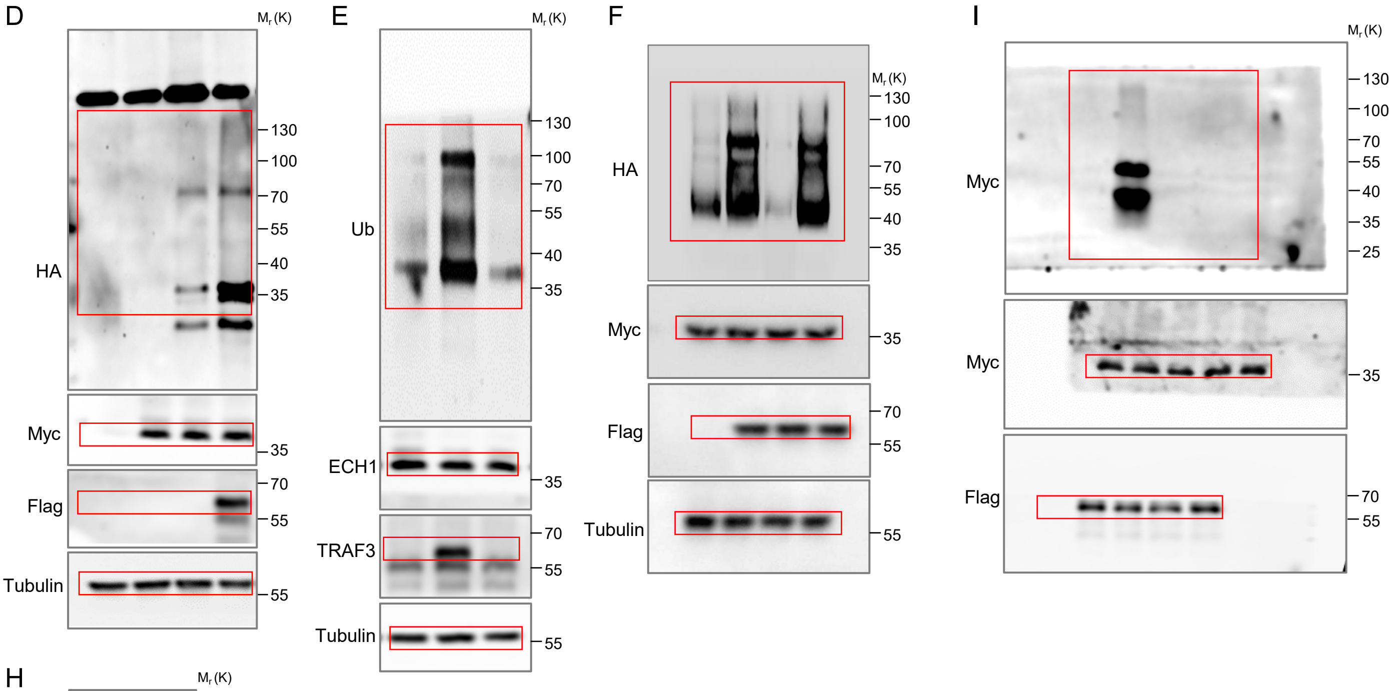

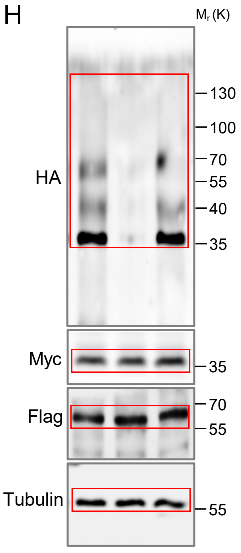

Figure 4

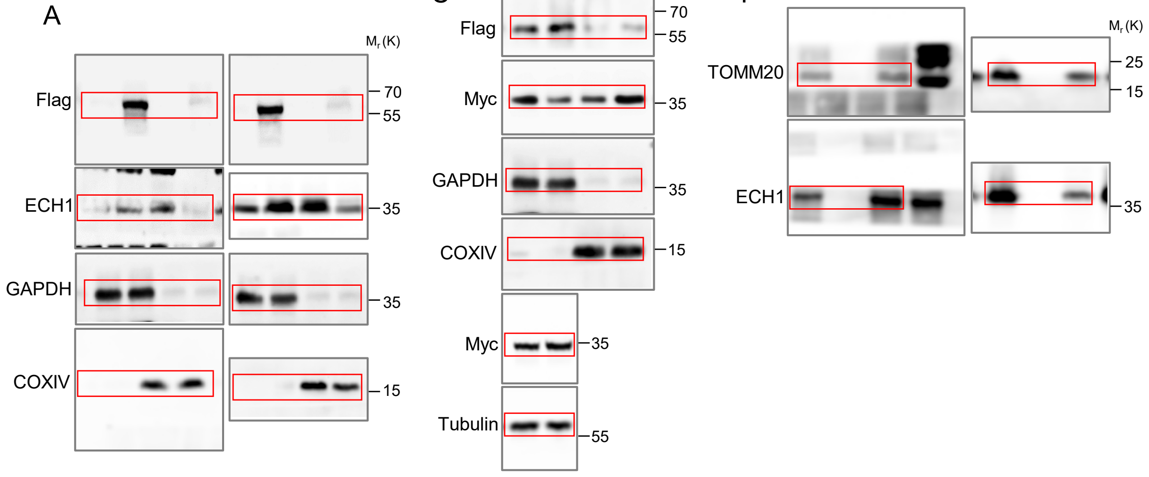

Figure 4

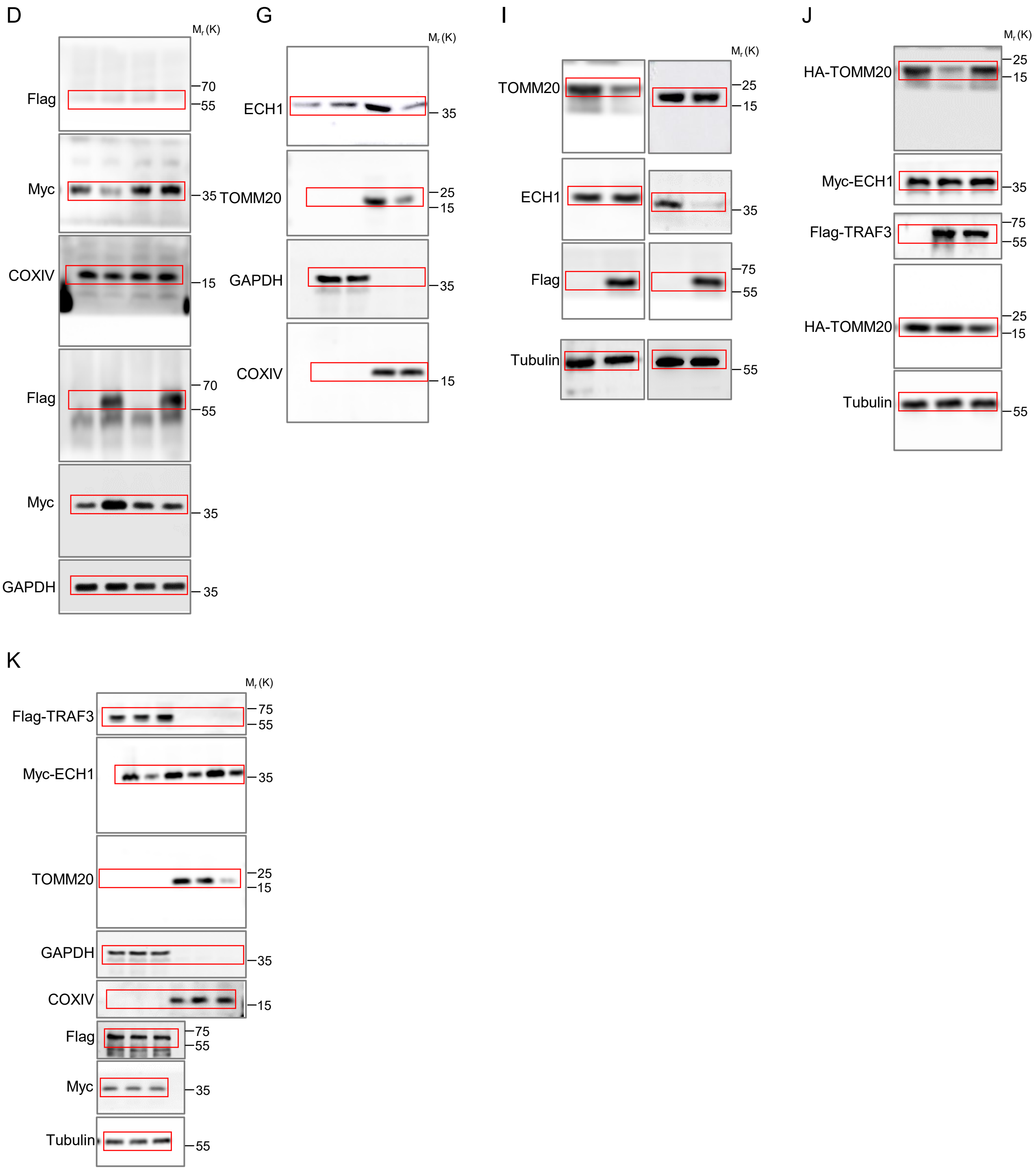

D
Mr (K)
Flag
70
55
Myc
35
COXIV
15
Flag
70
55
Myc
35
GAPDH
35
G
Mr (K)
ECH1
35
TOMM20
25
15
GAPDH
35
COXIV
15
I
Mr (K)
TOMM20
25
15
ECH1
35
Flag
75
55
Tubulin
55
J
Mr (K)
HA-TOMM20
25
15
Myc-ECH1
35
Flag-TRAF3
75
55
HA-TOMM20
25
15
Tubulin
55
K
Mr (K)
Flag-TRAF3
75
55
Myc-ECH1
35
TOMM20
25
15
GAPDH
35
COXIV
15
Flag
75
55
Myc
35
Tubulin
55

Supplementary Figure 1

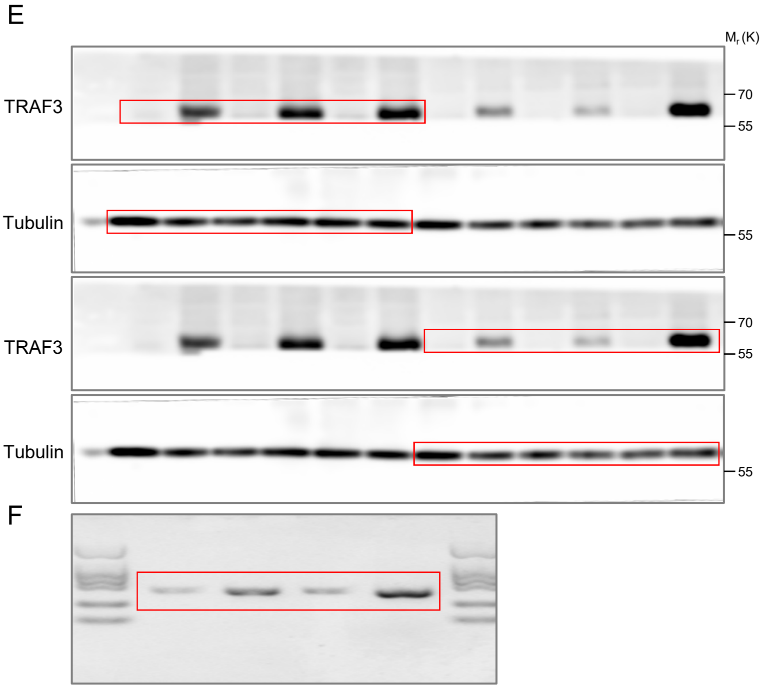

Supplementary Figure 2

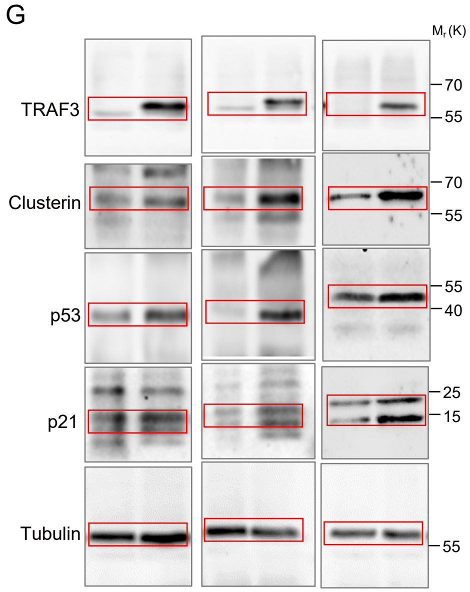

Supplementary Figure 3

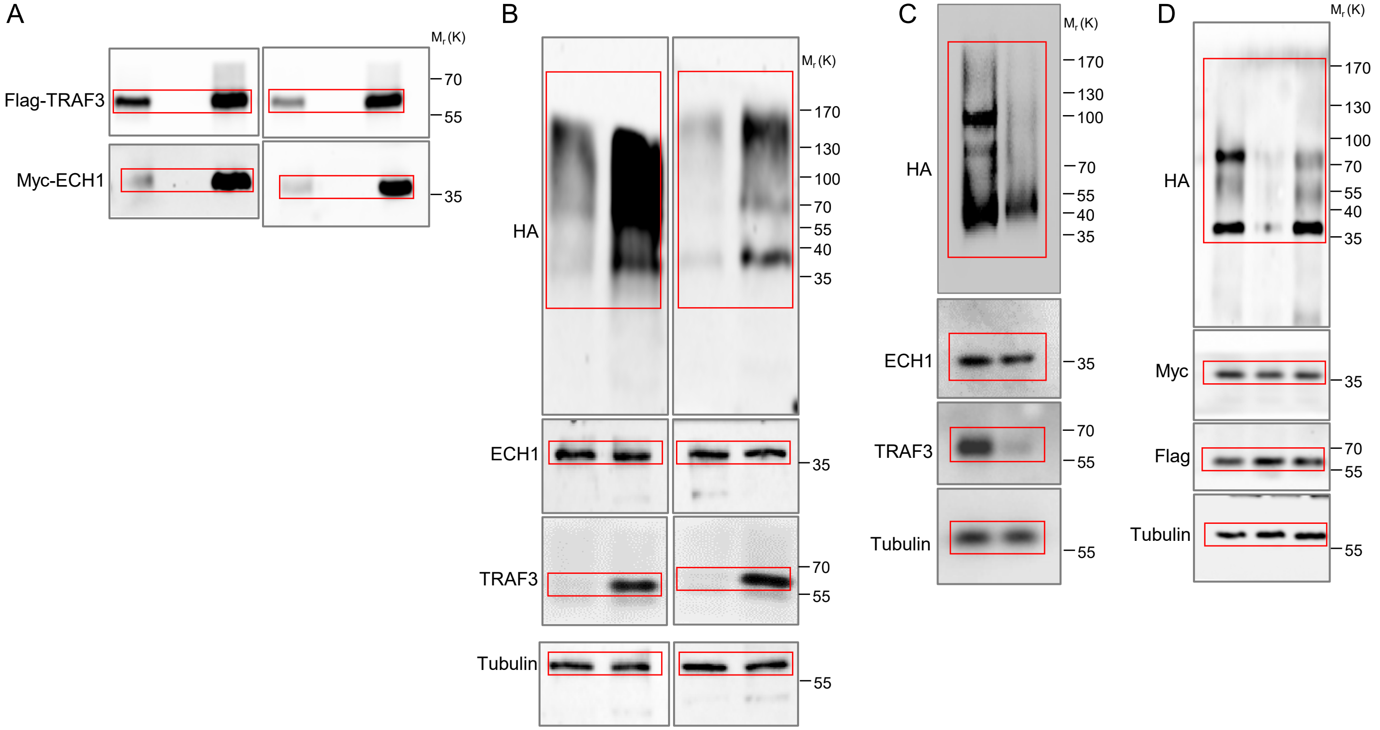

Supplementary Figure 3

E

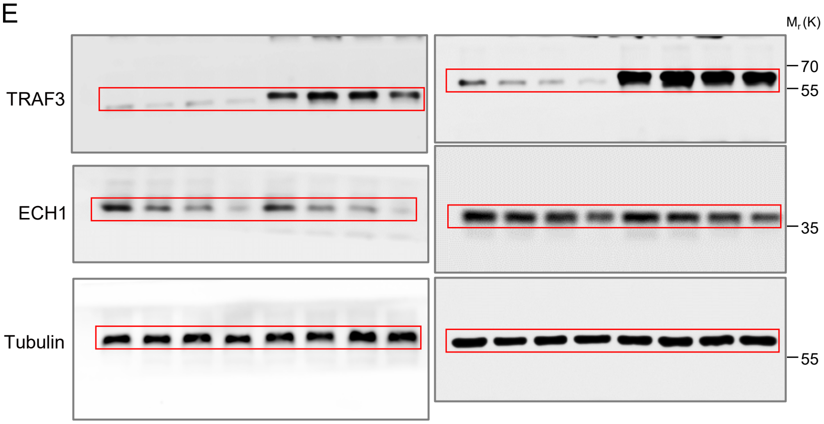

Supplementary Figure 4

A

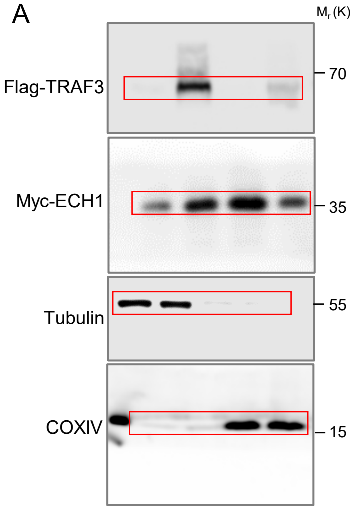

D

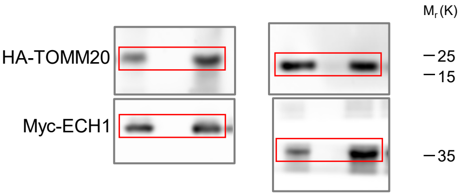

E

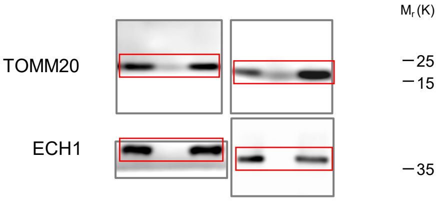

Supplementary Figure 6

F

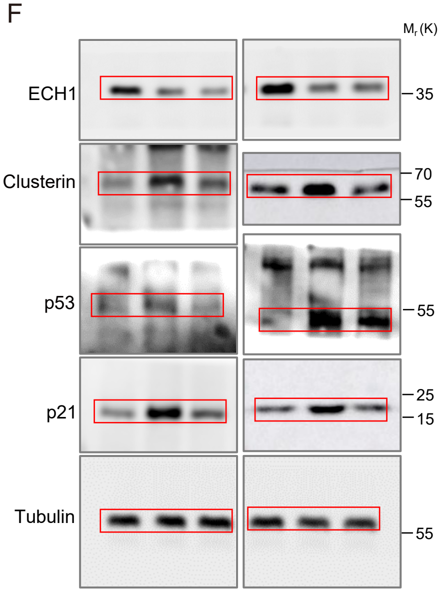

Supplementary Figure 7

B

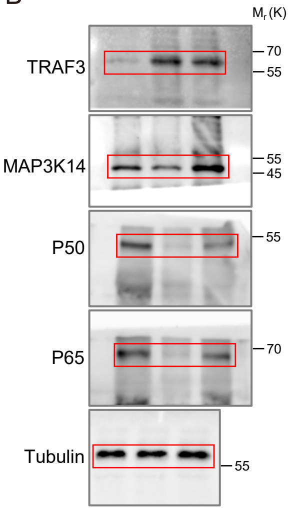

Supplementary Figure 8

F

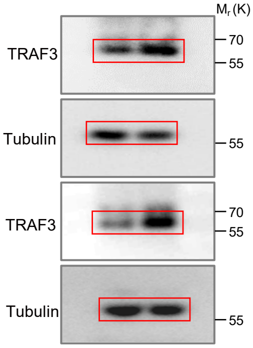

Supplement: Unedited blot and gel images [file jci-135-178550-s010.pdf]
